# Early preclinical experience of a mixed reality ultrasound system with active GUIDance for NEedle-based interventions: The GUIDE study

**DOI:** 10.1016/j.cvdhj.2022.07.072

**Published:** 2022-08-04

**Authors:** David Bloom, Jamie N. Colombo, Nathan Miller, Michael K. Southworth, Christopher Andrews, Alexander Henry, William B. Orr, Jonathan R. Silva, Jennifer N. Avari Silva

**Affiliations:** ∗ Division of Pediatric Cardiology, Department of Pediatrics, Washington University School of Medicine, St. Louis, Missouri; † Pediatric Electrophysiology Laboratory, St. Louis Children’s Hospital, St. Louis, Missouri; ‡ Sentiar, Inc., St. Louis, Missouri; § Department of Biomedical Engineering, Washington University McKelvey School of Engineering, St. Louis, Missouri

**Keywords:** Mixed reality, Tool tracking, Ultrasound, Vascular access, Vascular variant

## Abstract

**Background:**

Use of ultrasound (US) to facilitate vascular access has increased compared to landmark-based procedures despite ergonomic challenges and need for extrapolation of 2-dimensional images to understand needle position. The MantUS™ system (Sentiar, Inc.,) uses a mixed reality (MxR) interface to display US images and integrate real-time needle tracking.

**Objective:**

The purpose of this prospective preclinical study was to evaluate the feasibility and usability of MantUS in a simulated environment.

**Methods:**

Participants were recruited from pediatric cardiology and critical care. Access was obtained in 2 vascular access training models: a femoral access model and a head and neck model for a total of 4 vascular access sites under 2 conditions—conventional US and MantUS. Participants were randomized for order of completion. Videos were obtained, and quality of access including time required, repositions, number of attempts, and angle of approach were quantified.

**Results:**

Use of MantUS resulted in an overall reduction in number of needle repositions (*P* = .03) and improvement in quality of access as measured by distance (*P* <.0001) and angle of elevation (*P* = .006). These findings were even more evident in the right femoral vein (RFV) access site, which was a simulated anatomic variant with a deeper more oblique vascular course. Use of MantUS resulted in faster time to access (*P* = .04), fewer number of both access attempts (*P* = .02), and number of needle repositions (*P* <.0001) compared to conventional US. Postparticipant survey showed high levels of usability (87%) and a belief that MantUS may decrease adverse outcomes (73%) and failed access attempts (83%).

**Conclusion:**

Use of MantUS improved vascular access among all comers, including the quality of access. This improvement was even more notable in the vascular variant (RFV). MantUS readily benefited users by providing improved spatial understanding. Further development of MantUS will focus on improving user interface and experience, with larger clinical usage and in-human studies.

**Key Findings**

• MantUS™ improved participants’ ability to perform central vascular access and the quality of access attempts compared to conventional ultrasound alone. This was most evident in the anatomic variant model.
• Participants found MantUS easy to use, improved spatial awareness, and believed the technology would lead to reductions in failed access attempts and adverse events.
• Future development will focus on both hardware and software developments, including improvements to the user interface.
• For future studies to assess whether specific user groups derive greater benefit from MantUS, larger sample sizes will be required for adequately powering statistical analysis.

## Introduction

Ultrasound (US)-guided vascular access has become the standard of care for obtaining central vascular access (CVA).^1^ Use of US has led to a reduction in procedural complications and has improved accuracy compared to using anatomic landmarks alone.^2^^,^^3^ Current workflows for using US during vascular access requires a bimanual technique requiring users to position a US cart that supports a display within the room, often resulting in suboptimal viewing angles. This inevitably causes the user’s head to look away from his/her hands and the trajectory of the needle. The proceduralist must then mentally assimilate a 3-dimensional (3D) understanding of the vascular structures from the US image while simultaneously integrating the projected path of the needle into this model. Limitations of this protocol specifically relate to difficulty visualizing the needle tip relative to the target, projected needle path and ergonomics/body positioning of the physician.^4^ These limitations may be amplified in patients with anatomic variations of their vessels.

Medical extended reality technologies have rapidly evolved and have been adopted for a wide variety of use cases, spanning teaching, training, education, and most recently intraprocedural use for simple and complex medical procedures.^5^, ^6^, ^7^, ^8^ For example, the CommandEP™ mixed reality (MxR) system (Sentiar, Inc., St. Louis, MO) deployed on a Microsoft HoloLens (Microsoft Inc., Redmond, WA) headset worn during cardiac electrophysiological procedures provides the physician with a 3D digital visualization of patient cardiac anatomy with real-time catheter locations, as well as the ability to control these data in a hands-free manner.^5^^,^^9^ Early clinical studies have shown improvements in catheter navigation accuracy and improvements in procedural workflow.^10^^,^^11^ In interventional cardiology, MxR has been used in percutaneous coronary interventions,^12^ valve interventions,^13^^,^^14^ pulmonary artery interventions,^15^^,^^16^ and left atrial appendage occlusion,^17^^,^^18^ with promising early results.

MantUS™ (Sentiar, Inc.) , using the Microsoft HoloLens 2 (an MxR display), displays real-time US imaging and needle tracking with the goal of improving US-guided interventions. The display provides IS visualization in 2 duplicated views, with needle tracking displaying the anticipated needle trajectory and visual cues noting the proximity of the needle tip to the plane of the US. A hands-free interface allows the physician to interact with the data using a gaze–dwell interface.^11^^,^^19^ (For video demonstration, see https://www.youtube.com/watch?v=n1DggC8a7tc).

The aim of this study was to assess the feasibility and usability of MantUS during vascular access in a prospective preclinical study conducted in 2 simulated vascular access trainers, comparing user performance using conventional US vs MantUS. We hypothesized that (1) MantUS would improve accuracy and efficiency for central vascular access (CVA) for all comers (as measured by time to access, number of access attempts, and number of needle repositions); (2) MantUS may provide increased benefit for difficult access; and (3) the MantUS prototype would be usable.

## Methods

A prospective preclinical feasibility and usability study was designed and performed at Washington University School of Medicine/St. Louis Children’s Hospital, with enrolled pediatric physicians as study subjects. The study received approval from the Washington University School of Medicine Institutional Review Board. Informed consent was obtained from each study participant.

Study subjects were recruited from the Department of Pediatrics, Divisions of Pediatric Cardiology and Pediatric Critical Care at Washington University in St. Louis, given the clinical experience of using US to obtain CVA in the user group. A recruitment goal of 30 study subjects was targeted.

### MantUS system

**Figure 1:**
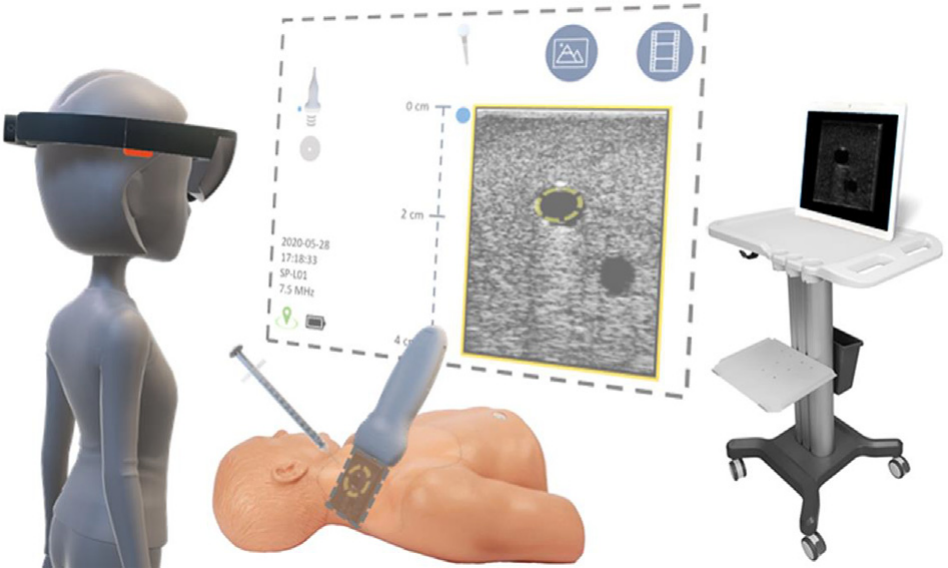
Graphic representation of the MantUS™ mixed reality (MxR) platform used during testing. The figure (left) represents a study participant wearing the MxR headset with the 2 duplicated ultrasound (US) views: (1) the “billboard view” **(top center)** or head-up display, which can be positioned anywhere in the room convenient for viewing; and (2) the “probe view” **(bottom center)**, which displays the US image at the tip of the probe to scale, allowing for a co-registered image overlying patient anatomy. A standard US screen display **(right)** also was available in the room.

**Figure 2:**
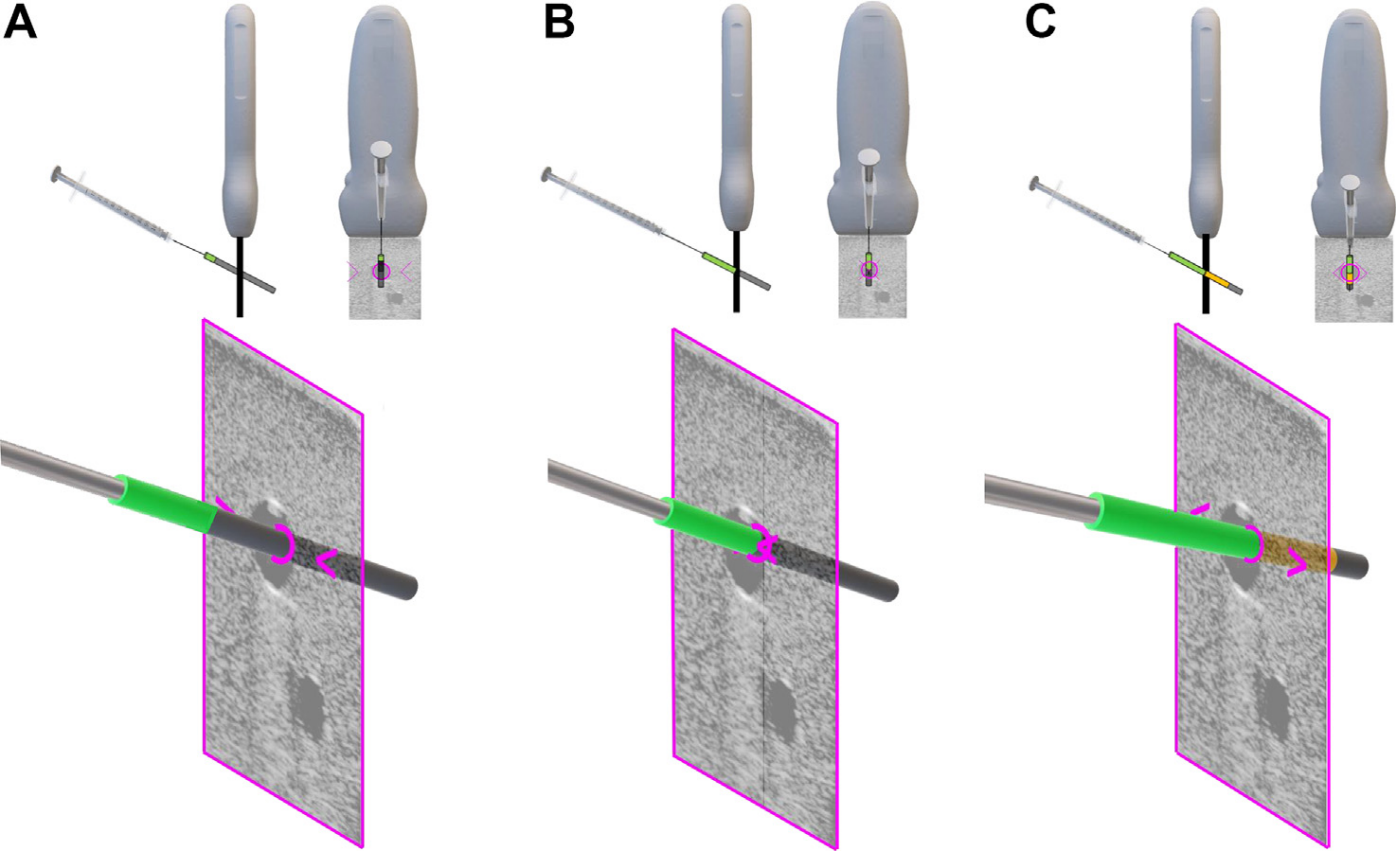
User interface for needle position. The display shows the anticipated trajectory of the needle in relation to the tip of the needle to the ultrasound (US) plane. **A:** The anticipated path of the needle is displayed as a gray cylindrical line, with the green cylinder meaning the needle is approaching the plane of the US. The lateral chevrons move toward each other as the tip approaches the plane of the US. **B:** As the needle tip is close to intersection with the ultrasound plane, the green cylinder and chevrons will intersect in the center of the circle. **C:** As the needle tip passes the US plane, the cylinder becomes gold and the chevrons move away from each other.

The MantUS system is composed of 4 components: (1) a MantUS proprietary US probe; (2) a head-mounted MxR display (Microsoft HoloLens 2); (3) integrated proprietary needle tracking software; (4) a syringe-mounted tracking sensor. US images are displayed in both the “billboard” view and “probe-tip” view and provide the user with a hands-free interface to display the image in a comfortable viewing position (Figure 1). In both views, tool tracking visualization displays the anticipated needle trajectory, as well as the proximity of the needle tip to the US plane. The anticipated path of the needle is displayed as a gray cylindrical line. The needle tip proximity to the US plane is displayed (1) on the gray line using color change representing crossing the plane (green = approaching the plane of the US; orange = past the plane of the US); and (2) by lateral chevrons that move toward each other as the tip approaches the plane of the US; chevrons move away from each other when the tip is past the plane of the US (Figure 2).

### Study protocol

Before the MantUS was used, all study participants completed a demographic form detailing their medical specialty, career stage, and frequency of access during their routine clinical practice. Next, participants completed a brief, scripted orientation to the system and study design. Participants then had the opportunity to practice using both the conventional US system and the MantUS system on a vascular US training phantom (CAE Healthcare, Montreal, Canada). Training time was unlimited and dictated by user preference.

Subjects completed study tasks under 2 randomized conditions: (1) conventional US first or (2) MantUS first. Study tasks were performed on 2 vascular access trainers: an upper torso model (CAE Blue Phantom Gen II Torso Central Line and Regional Anesthesia Ultrasound trainer, CAE Healthcare, Montreal, Canada) and a femoral vascular access model (CAE Blue Phantom Gen II Femoral Access and Regional Anesthesia Ultrasound trainer, CAE Healthcare) with vascular access obtained in the right internal jugular vein, right carotid artery, right femoral vein (RFV), and right femoral artery (RFA). Order of access sites was fixed for each participant.

Each study task was video-recorded using a Logitech Pro Webcam (Logitech International SA, San Jose, CA) to allow for complete data review and analysis postprocedure with all participant identifiers removed. Only the study subject’s hands, vascular access trainer, and syringe/needle were recorded. Participants were not time-restricted during study tasks. Outcomes measured included time to access, number of access attempts, and number of needle repositions. Time to access was measured starting with placement of the US probe on the training phantom to completion once there was free-flowing contrast (consistent with access site) in the syringe. Number of access attempts was defined as the number of times the percutaneous needle punctured the training phantom (pulling needle back to skin and readvancing counted as additional attempt). Number of needle repositions was quantified by counting the times a noticeable change was made in either the needle trajectory or the angle of approach. After completion of all tasks, all participants completed a 13-question exit survey using a 5-point Likert scale to assess usability. Each video was reviewed and adjudicated by 2 study team members to document time to access (average time between adjudicators), number of access attempts, and number of needle repositions (consensus between adjudicators).

### Quality of access

**Figure 3:**
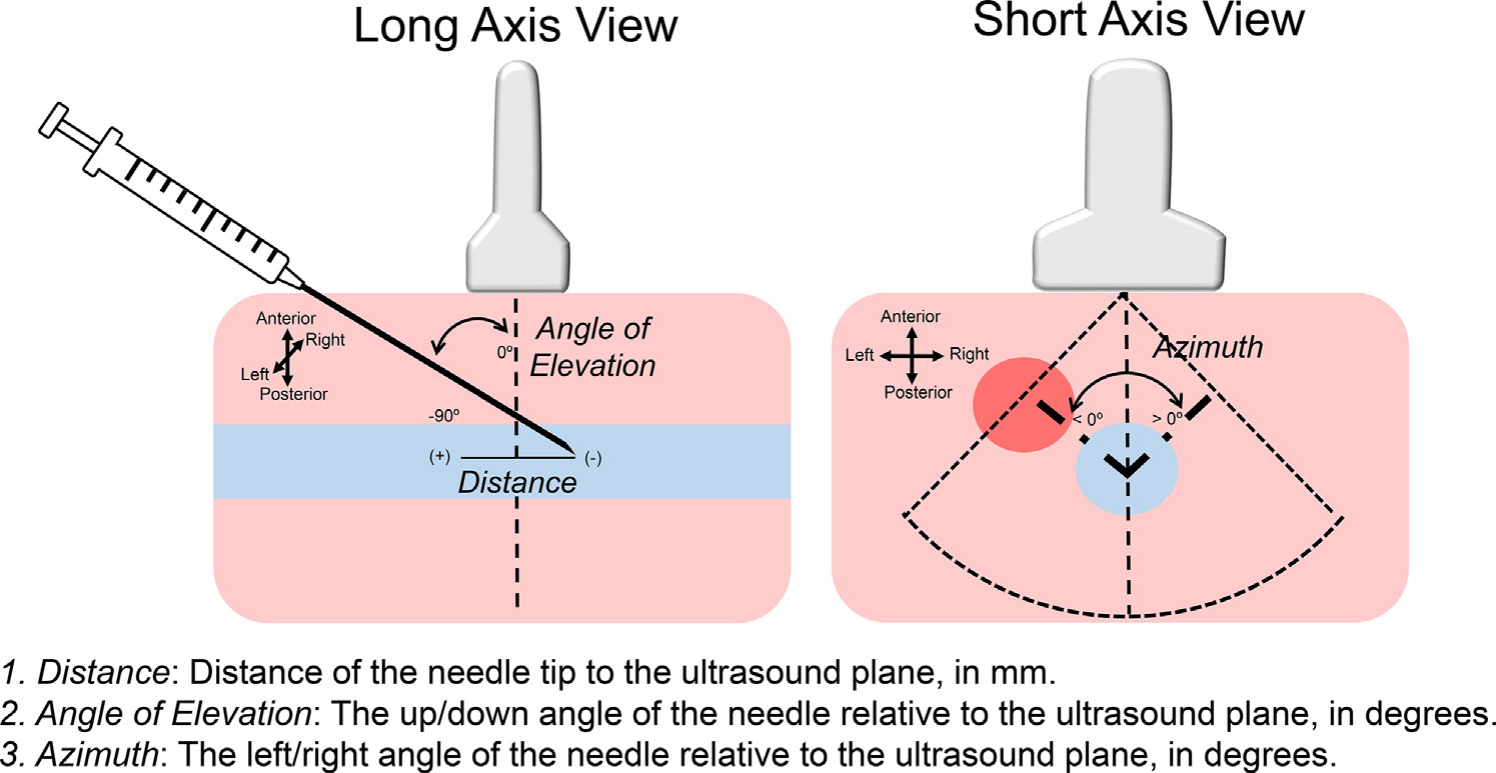
Markers of quality of access. Three discrete measurements were used to evaluate quality of access, shown in this graphic. **Left:** Long-axis view of the vessel. Both angle of elevation and distance are displayed. (1) Distance describes the distance of the needle tip entry into the blood vessel from the plane of the US. A positive value (+) refers to the tip approaching the US plane, and a negative value (–) means the tip has gone past the US plane. (2) Angle of elevation is the up/down angle of the needle relative to the US plane, in degrees. A value of 0° means the needle is parallel to the US plane, and –90° means the needle is perpendicular to the US plane. **Right:** Azimuth, a measurement of laterality (left/right) for needle entry relative to the vessel in the plane of the US, also measured in degrees. A value of 0° indicates the needle tip is perpendicular to the US plane. A negative value indicates the plunger is to the left of the needle tip, whereas a positive value means the plunger is to the right of the needle tip.

Needle tracking data were downloaded and analyzed offline poststudy to assess the quality of the access. Three parameters were collected from these data to define quality of access: distance, angle of elevation, and azimuth (Figure 3). Distance describes the distance from the needle tip entry into the blood vessel from the plane of the US, as measured in millimeters. Conceptually, this measurement reflects the user’s ability to understand the 3D relationship of the US image and the needle. A positive measurement of distance refers to the tip approaching the US plane, and a negative value means the tip has gone past the US plane. Angle of elevation of the needle from the plane of the US image (up/down angle of the needle relative to the US plane) is measured in degrees. A value of 0 means the needle is parallel to the US plane, and –90 means the needle is perpendicular to the US plane. Previous results published by other investigators suggest that an angle of entry of 30°–45° for vascular access is ideal.^20^, ^21^, ^22^ Azimuth is the measurement of laterality (left/right) of entry of the needle relative to vessel in the US plane, also measured in degrees. A value of 0 indicates the needle tip and plunger are directly perpendicular to the US plane; a negative value indicates the plunger on the syringe is positioned to the left of the needle tip and a positive value indicates the plunger is positioned to the right of the needle tip. Ideal vascular access attempts would have an azimuth close to 0 when viewing the vessel in a short-axis projection (Figure 3).

### Statistical analysis

Data analysis was performed using Microsoft Excel (Microsoft Inc.) and GraphPad Prism Version 9 (GraphPad Software, San Diego, CA). Descriptive data are presented as average with standard deviation or percentage as appropriate. Analyses included paired Student *t* tests and analysis of variance where appropriate. Violin plots were generated in GraphPad with truncated plots used as appropriate. Statistical significance was set for *P* <.05.

## Results

### Study subject demographics

A total of 30 study subjects were enrolled in the study, with 57% pediatric cardiologists (n = 17/30), 27% pediatric intensivists (n = 8/30), and 17% dual-trained pediatric cardiac intensivists (n = 5/20). When grouped by career stage, 60% of study subjects were fellows in training, 23% were attendings ≤5 years into practice, 7% were attendings between 6 and 10 years of practice, and 10% were attendings >10 years into practice. Lastly, when grouped by frequency of access, 37% infrequently obtained vascular access during their routine clinical workload (<4 times per month), 20% obtained vascular access often (>4 times per month), and 43% obtained vascular access frequently (>3 times per week).

### Training data

The slide orientation was administered by the same individual for each participant, with participants spending an average of 11 ± 1 minutes). For the hands-on practice before initiation of study, users spent an average of 6 ± 7 minutes, with 97% (n = 29/30) practicing only on the MantUS system.

### All-comer data

**Figure 4:**
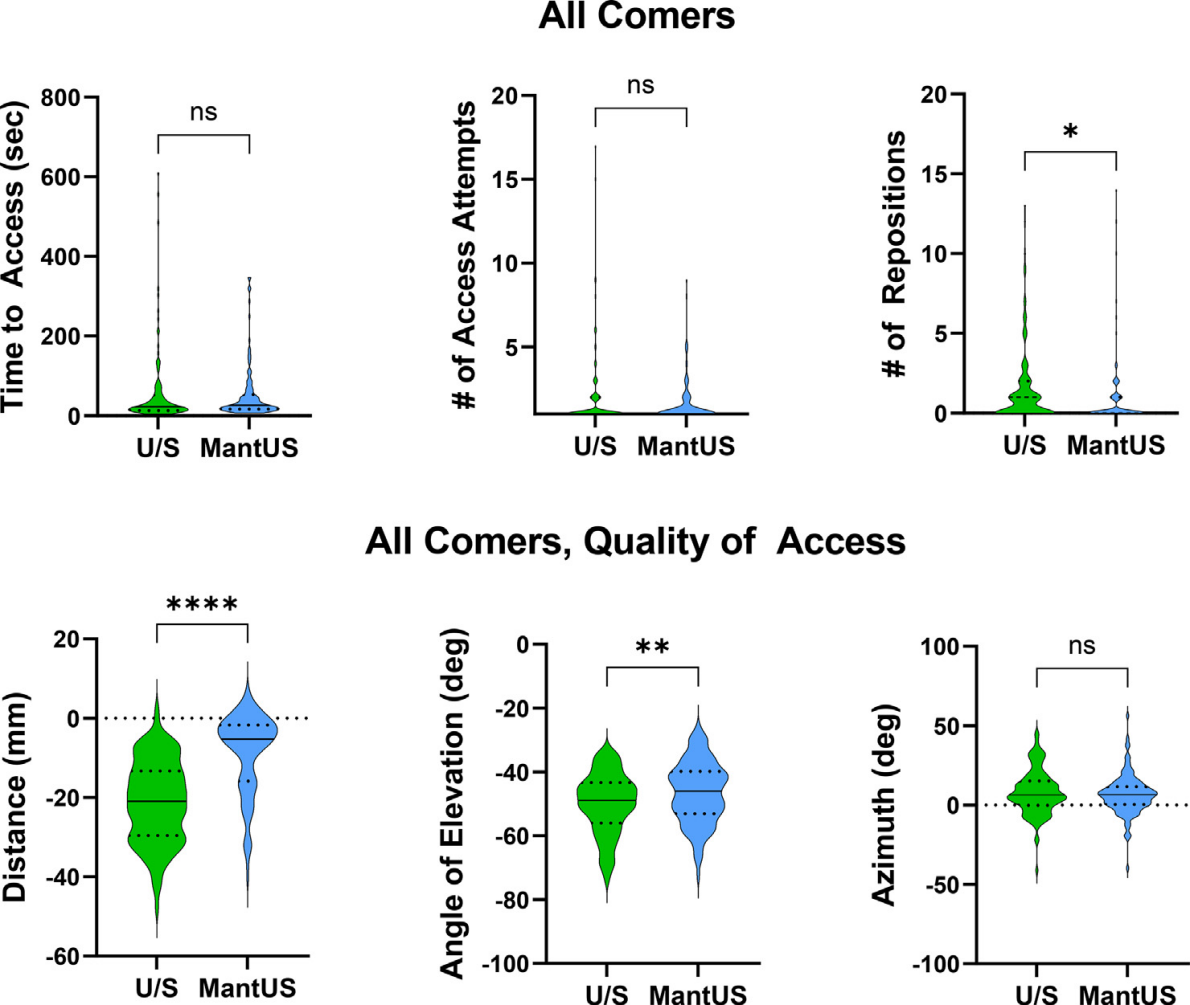
Violin plot for all comers. **Top row:** Violin plot shows that there was no significant difference between conventional ultrasound (U/S) and MantUS™ with regard to access or number of access attempts. There was a significant reduction in number of needle repositions when MantUS was used (*P* = .03). **Bottom row:** For quality of access, there was an improvement in distance (*P* <.0001) and angle of elevation (*P* = .006) when MantUS was used but no significant difference in azimuth (*P* = ns) between the 2 methods. ∗*P* ≤ .05, ∗∗*P* ≤ .01, ∗∗∗∗*P* ≤ .0001.

There was no significant difference in time to access when using conventional US (59 ± 100 seconds) vs MantUS (54 ± 71 seconds) (*P* = .6). There were fewer number of access attempts when using MantUS (conventional US 2 ± 2 vs MantUS 1.5 ± 1; *P* = .08) and even fewer number of needle repositions when using MantUS (conventional US 1.5 ± 3 vs MantUS 0.8 ± 2; *P* = .03) (Figure 4).

When assessing all-comer quality of access, distance of access relative to the US plane using conventional US was –21±10 mm vs MantUS 8 ± 10 mm (*P* <.001). The angle of elevation using conventional US was –50° ± 10° vs MantUS –46° ± 10° (*P* = .002). Azimuth measurements were similar for conventional US (8° ± 14°) vs MantUS (7° ± 12°; *P* = .3) (Figure 4). Please see Supplemental Data.

### Survey results

**Figure 5:**
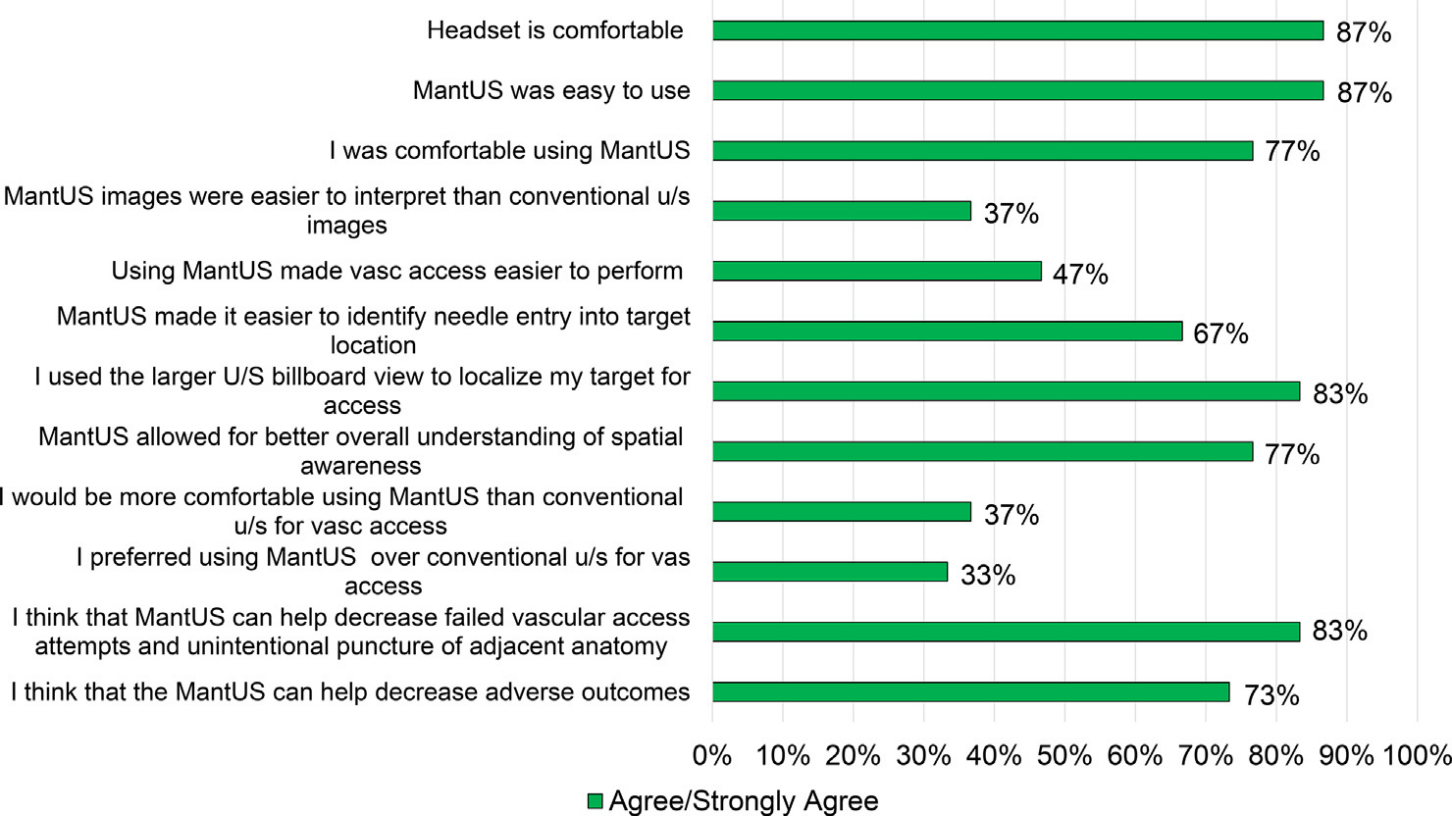
Results of a 13-question survey completed by each study participant at the end of his/her experience using MantUS™. Responses of agree/strongly agree are displayed in *green*. U/S = ultrasound.

Study participants agreed/strongly agreed that MantUS was easy to use, and that the head mounted display was comfortable to wear (87%), with 77% of participants being comfortable using MantUS. Most users (83%) used the larger “billboard” view to localize the vascular access target, with 83% of users feeling that MantUS allowed for better overall understanding of spatial awareness. Overall, 83% of study participants thought that MantUS can help decrease failed vascular access attempts and unintentional punctures of adjacent anatomies, and 73% thought that MantUS can help decrease adverse outcomes (Figure 5). Of the study subjects, 37% felt that MantUS images were easier to interpret than conventional US images, with 37% being more comfortable with MantUS over conventional US for vascular access, and 47% felt that MantUS made vascular access easier to perform (Figure 5).

### Subgroup analysis: Vascular anatomic variant access

**Figure 6:**
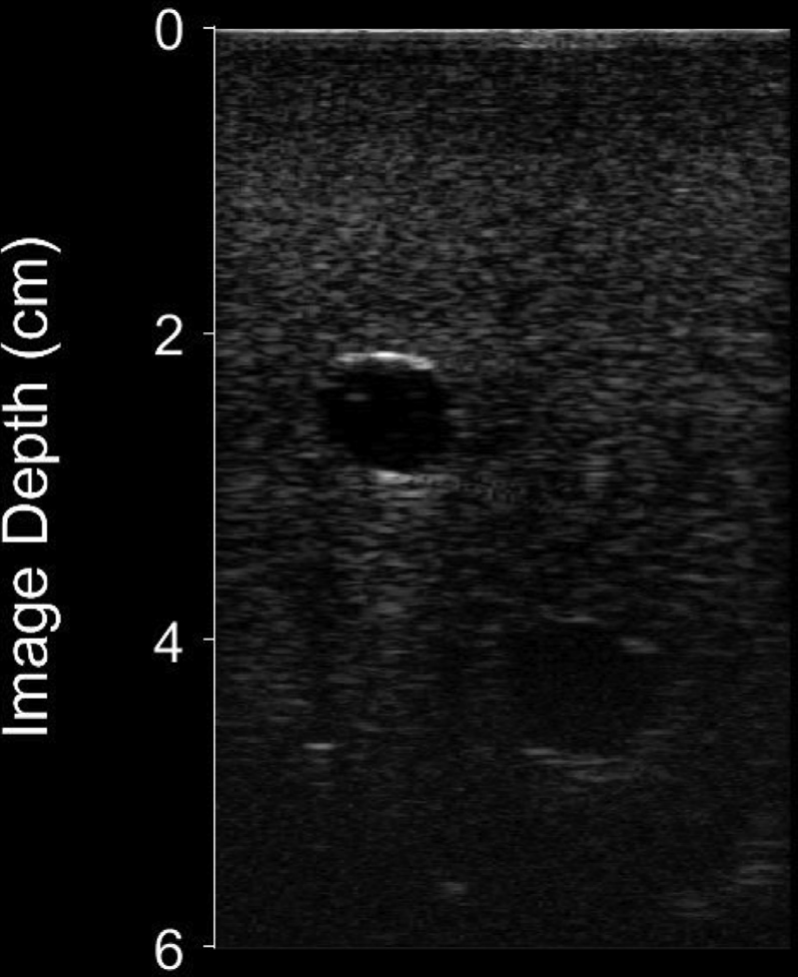
Static short-axis ultrasound image from the femoral vascular access model showing the right femoral artery (RFA) **(left)** and right femoral vein (RFV) **(right)**. The RFV is notably 2 cm deeper than the RFA.

**Figure 7:**
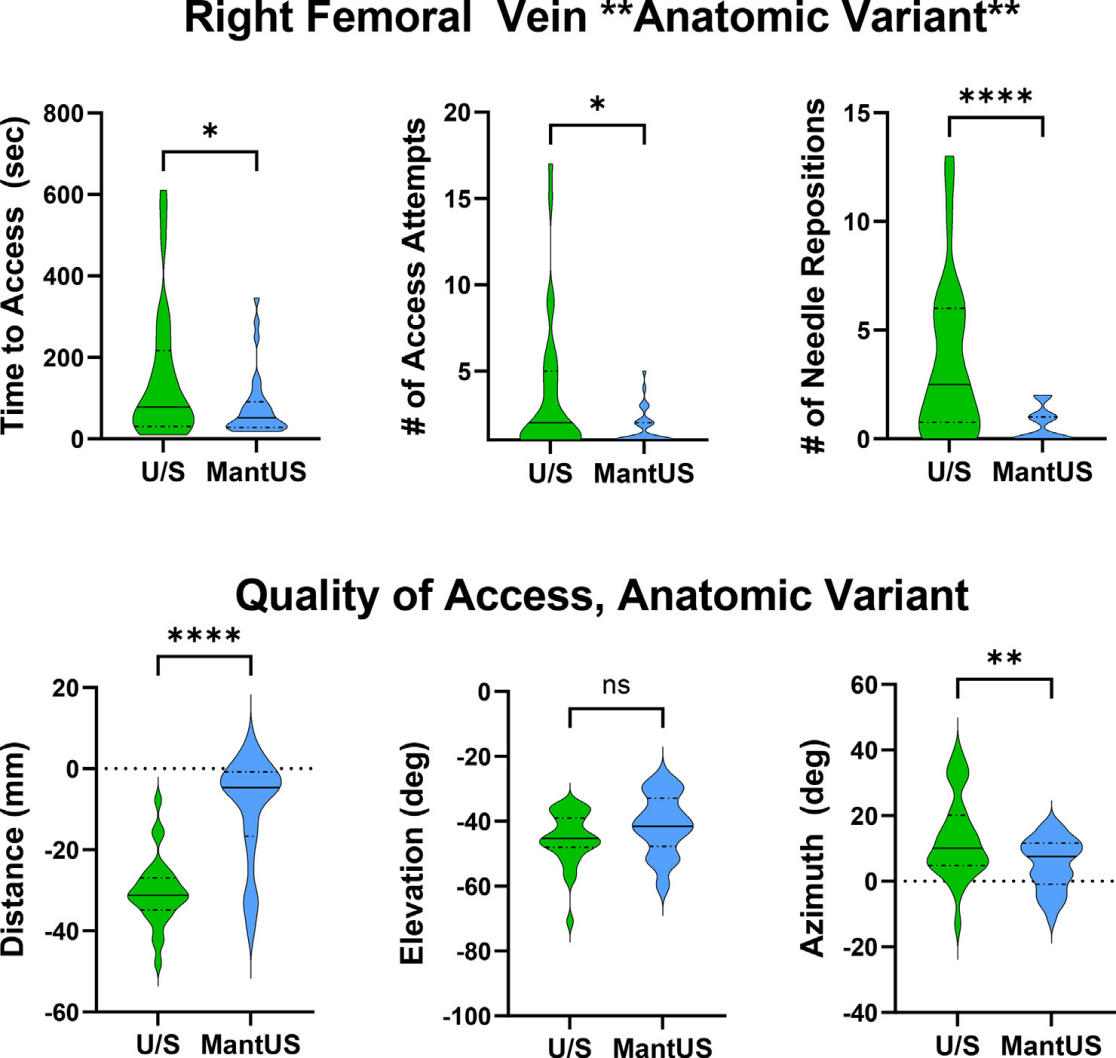
Violin plot for right femoral venous ∗∗Anatomic Variant∗∗ (right femoral vein) access. **Top row:** Significant improvement is seen in time to access (*P* = .04), number of access attempts (*P* = .02), and number of needle repositions (*P* <.0001) when using MantUS™. **Bottom row**: When assessing quality of access, there was improvement in distance (*P* <.0001) and azimuth (*P* = .007) when using MantUS™ but no significant difference in angle of elevation (*P* = ns). ∗*P* ≤ .05, ∗∗*P* ≤ .01, ∗∗∗∗*P* ≤ .0001.

The RFV access site was an anatomic variant in the vascular access trainer, with the vessel displaced inferiorly compared to the RFA (Figure 6). When accessing the RFV, there was a significantly faster time to access using MantUS (conventional US 149 ± 163 seconds vs MantUS 80 ± 81 seconds; *P* = .04), with fewer number of access attempts (conventional US 4 ± 4 vs MantUS 1.6 ± 1; *P* = .02) and fewer number of needle repositions (conventional US 3.5 ± 3.6 vs MantUS 0.6 ± 0.8 seconds; *P* = .0001). When assessing quality of access, there was improved distance using MantUS (conventional US –30 ± 8 mm vs MantUS –10 ± 13 mm; *P* <.0001). There was no significant difference in angle of elevation (conventional US –45° ± 8° vs MantUS –41° ± 9°; *P* = .1). There was improvement in azimuth using MantUS (conventional US 13° ± 12° vs MantUS 5° ± 8°; *P* = .008) (Figure 7).

## Discussion

There were several novel findings from this first description of MantUS, an MxR US system with integrated tool tracking in a preclinical setting for vascular access. All-comer analysis showed a demonstrable benefit using MantUS for vascular access in both number of needle repositions and quality of access. The benefit of MantUS was further amplified when performing vascular access in an anatomic variant (in this case, femoral venous access). Quantification of quality of access may be an important metric, particularly if these data are displayed in real time. Lastly, the system was user-friendly, although there is room for continued interface development and improvement.

Previous data have shown that adverse outcomes during vascular access and central venous catheter placement, such as infection, thrombosis, inadvertent puncture of adjacent vasculature, and vessel injury, are associated with provider experience, multiple access attempts, and needle repositions.^23^, ^24^, ^25^ The adoption of US over landmark technique has improved rates of successful vascular access by 14%–29% and reduced inadvertent vascular puncture by 7.7%.^26^ Data from the GUIDE study show that MantUS further improved the users’ ability to obtain vascular access compared to conventional US, showing statistical significance for reduction of needle repositions. This study may have been underpowered to demonstrate differences in time to access and number of access attempts.

Vascular anatomic variants are seen in 8%–36% of patients^27^, ^28^, ^29^ and cannot be appreciated via traditional landmark access techniques for vascular access. Use of US for vascular access in patients with vascular variants has been shown to improve access rates.^29^ In this protocol, we were able to recapitulate an anatomic variant with RFV access site, as it took a markedly deep and oblique course relative to the RFA. The GUIDE study demonstrated that using MantUS further reduced time to access, number of access attempts, and number of needle repositions compared to conventional US. One can postulate that during the vascular access variant, which was a more challenging access site, participants cognitively relied on real-time needle tracking, which lead to improved results over conventional US. This finding will have important clinical implications moving forward, as anatomic vascular variants are more commonly described in older patients and females.^30^ Patients with congenital heart disease and those who previously have undergone catheterizations also may have vascular access challenges.^31^ In these patients, MantUS may be able to provide real-time feedback to the user, which may reduce time to access and unnecessary needle sticks and repositions, thereby limiting adverse outcomes.

Assessing quality of access is an important additive functionality of MantUS. In the GUIDE study, quality of access measures included distance, angle of elevation, and azimuth, which were assessed and evaluated poststudy. Although optimal angle of elevation previously has been defined as 30°–45°,^20^, ^21^, ^22^ we would add (1) distance ∼0 mm and (2) azimuth ∼0° when viewing the vessel in a short-axis projection. The measurement of distance provides useful insight into the user’s depth perception and 3D spatial awareness. Increasing the user’s ability to access a structure closer to the US plane (distance 0 mm being at the US plane itself) may lead to safer vascular access and decrease unintentional punctures of adjacent anatomies. MantUS led to statistically significant improvement in distance (closer to 0 mm) for all comers, as well as for each site-specific vascular access target compared to conventional US. Azimuth measurements can help the user perform a more central access to the structure, thereby limiting lateral access and potential endothelial injury. By providing this information to the user, a more optimal, central access can be targeted. MantUS did allow all users to perform a more central access (azimuth closer to 0°). MantUS proved to be capable of providing a quantitative assessment of quality of access, which may lead to safer and more optimal access.

Dynamic needle tip positioning is a modified US technique that combines the advantage of both the short-axis, out-of-plane view (optimal for visualization of relevant structures, such as vascular anatomy, nerves, and vessels) with long-axis, in-plane view (visualization of the position of the needle as it enters the vessel lumen) for vascular access.^32^ This technique requires active movement of the US probe to “follow the tip” of the needle and has been shown to improve outcomes.^32^^,^^33^ Given the ability of the MantUS interface to provide tool tracking registered to the US image, it may provide equivalent information to dynamic needle tip positioning with the added benefit of ease of use and limited US repositioning.

User experience measured by the survey can be divided into 3 categories: (1) comfort and ease of use; (2) image quality and spatial understanding; and (3) clinical applicability. From the perspective of comfort and ease of use, users rated MantUS highly. Areas for improvement were noted in both image quality/spatial awareness and clinical applicability. Despite most study subjects concluding that conventional US images were easier to interpret and stating increased comfort using conventional US, 77% of study subjects felt that MantUS improved their spatial understanding by making it easier to identify needle entry into the target location. Participants likely had difficulty using the “probe view” US image because it is quite small in size and may have obscured visualization of needle entry into the skin. This can be addressed by revising and optimizing the “probe view” in future studies. However, most study subjects believed that MantUS can help decrease failed vascular access attempts, unintentional puncture of adjacent anatomy, and adverse outcomes in vascular access procedures. Physicians were comfortable learning about and using innovative technology in a commonly performed procedure and appreciated the potential clinical benefit of MxR in daily procedures. Increasing training before use of the MantUS system coupled with improvements to hardware, software, and user interface likely will address user concerns regarding comfort and preference for conventional US.

### Study limitations

Study limitations can be grouped into MantUS limitations, protocol limitations, and phantom limitations. MantUS limitations include the use of an alpha prototype, which had unforeseeable technical glitches that could impact performance. Removal of smartphones, smartwatches, and magnetic ID/credit cards from users helped minimize these technical challenges. Image quality and latency occasionally required troubleshooting. Future versions would benefit from increased hands-free control of the image, including depth and gain. Protocol limitations included the addition of training materials, as well as a fixed protocol for obtaining access. This may have led to study participants “learning” strategies that confounded results, particularly for the last access site. Phantom vascular access models also have inherent limitations, including difficulty piercing the skin and air entry into tubing creating bubbles in the vasculature (creating bubble contrast on US). This was most evident in the arterial systems (right carotid artery and RFA) where significant bubble contrast was noted.

## Conclusion

This early feasibility study showed that MantUS improved all-comers’ ability to perform vascular access and improved the quality of access. This benefit was most evident in the vascular anatomic variant, with reduction in time to access, number of access attempts, and number of needle repositions, as well as improvement in quality of access. Quality-of-access parameters now can be quantified, including angle of elevation, distance, and azimuth. Participants found MantUS easy to use and believe it will lead to reductions in adverse outcomes during vascular access procedures and in failed access attempts. Future large-scale clinical trials with updated hardware and software are indicated to further test the value of this MxR technology.
