# Supplementary material for: Early preclinical experience of a mixed reality ultrasound system with active GUIDance for NEedle-based interventions: The GUIDE study
Source: Cardiovasc Digit Health J. 2022 Aug 4;3(5):232–40. doi: 10.1016/j.cvdhj.2022.07.072 (PMC9596321; doi:10.1016/j.cvdhj.2022.07.072)
Supplement: Supplemental Tables and Figures [file mmc1.pptx]

## Slide 1
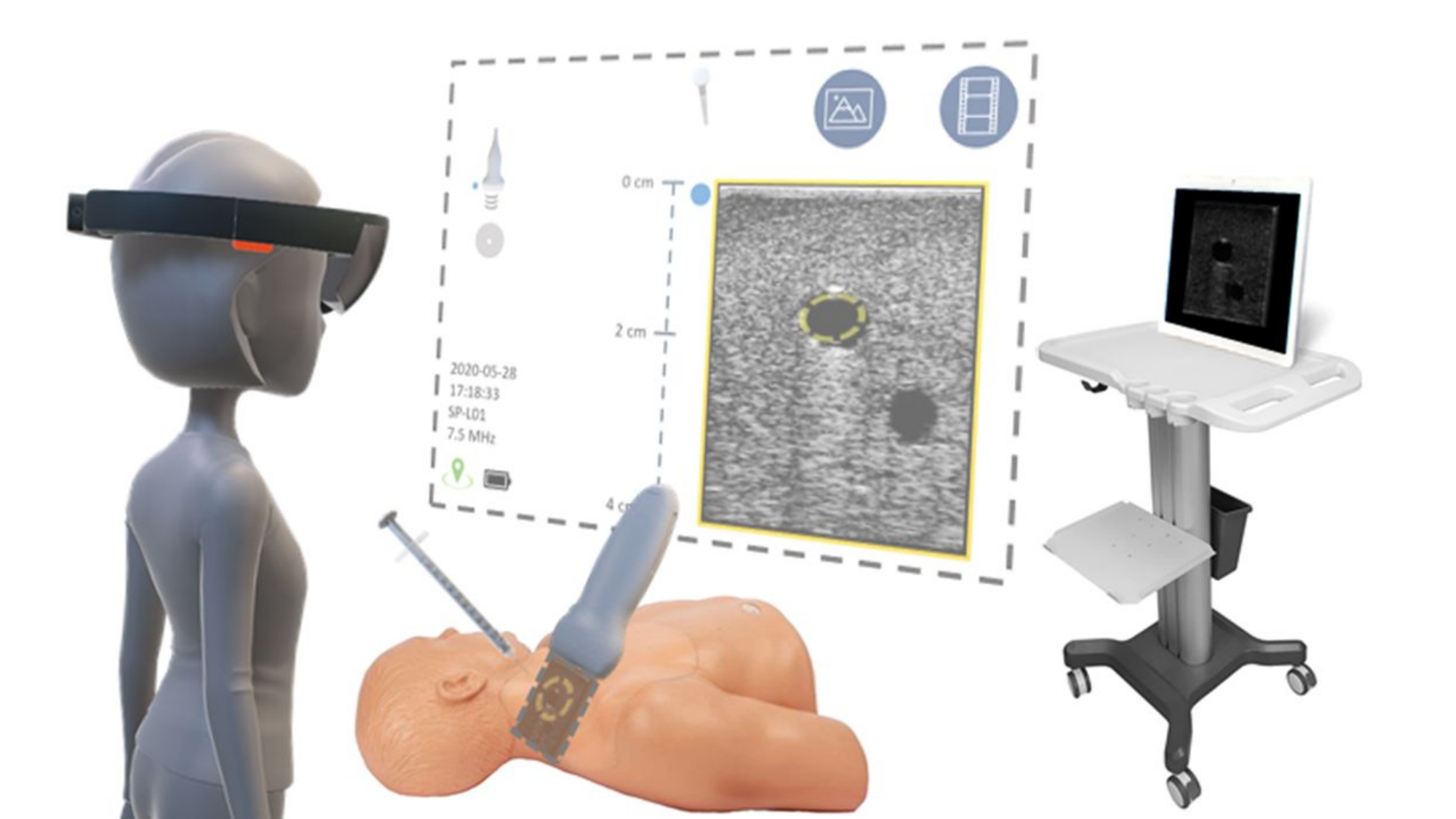

## Slide 2
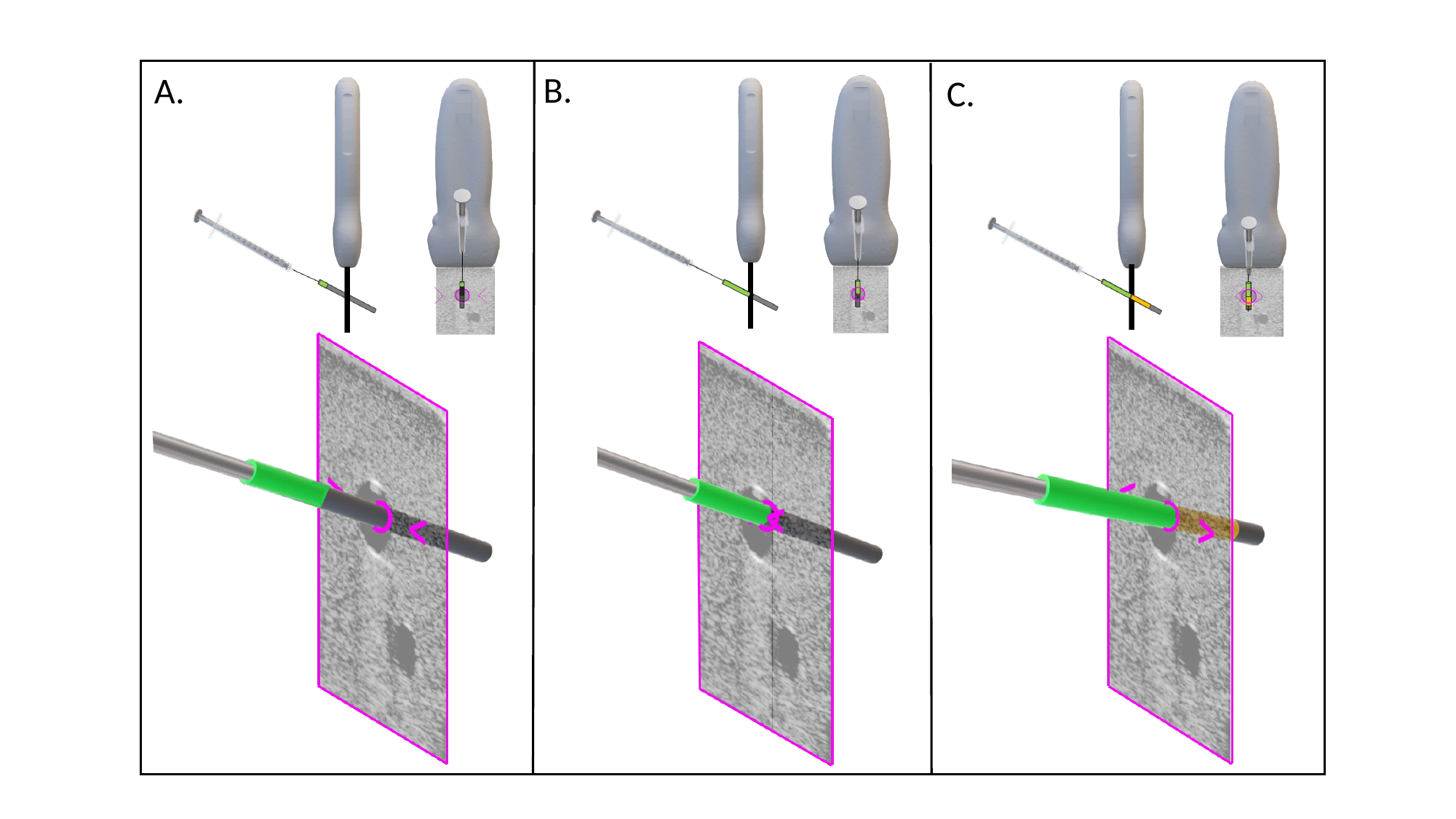

B.
A.
C.

## Slide 3
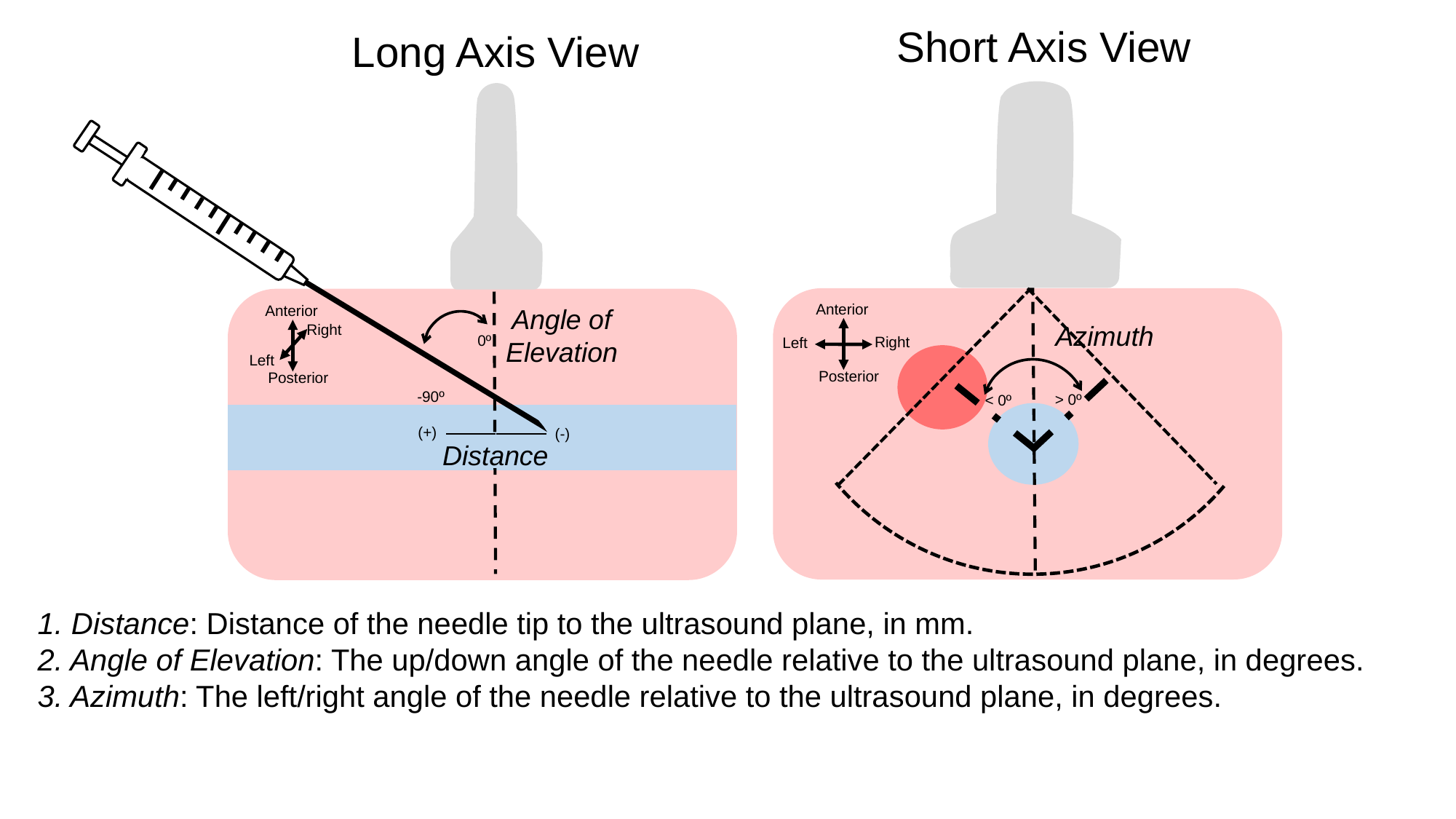

Short Axis View
Anterior
Azimuth
Left
Right
Posterior
Long Axis View
Anterior
Angle of Elevation
Right
0º
Left
Posterior
-90º
> 0º
< 0º
(+)
(-)
Distance
1. Distance: Distance of the needle tip to the ultrasound plane, in mm.
2. Angle of Elevation: The up/down angle of the needle relative to the ultrasound plane, in degrees.
3. Azimuth: The left/right angle of the needle relative to the ultrasound plane, in degrees.

## Slide 4
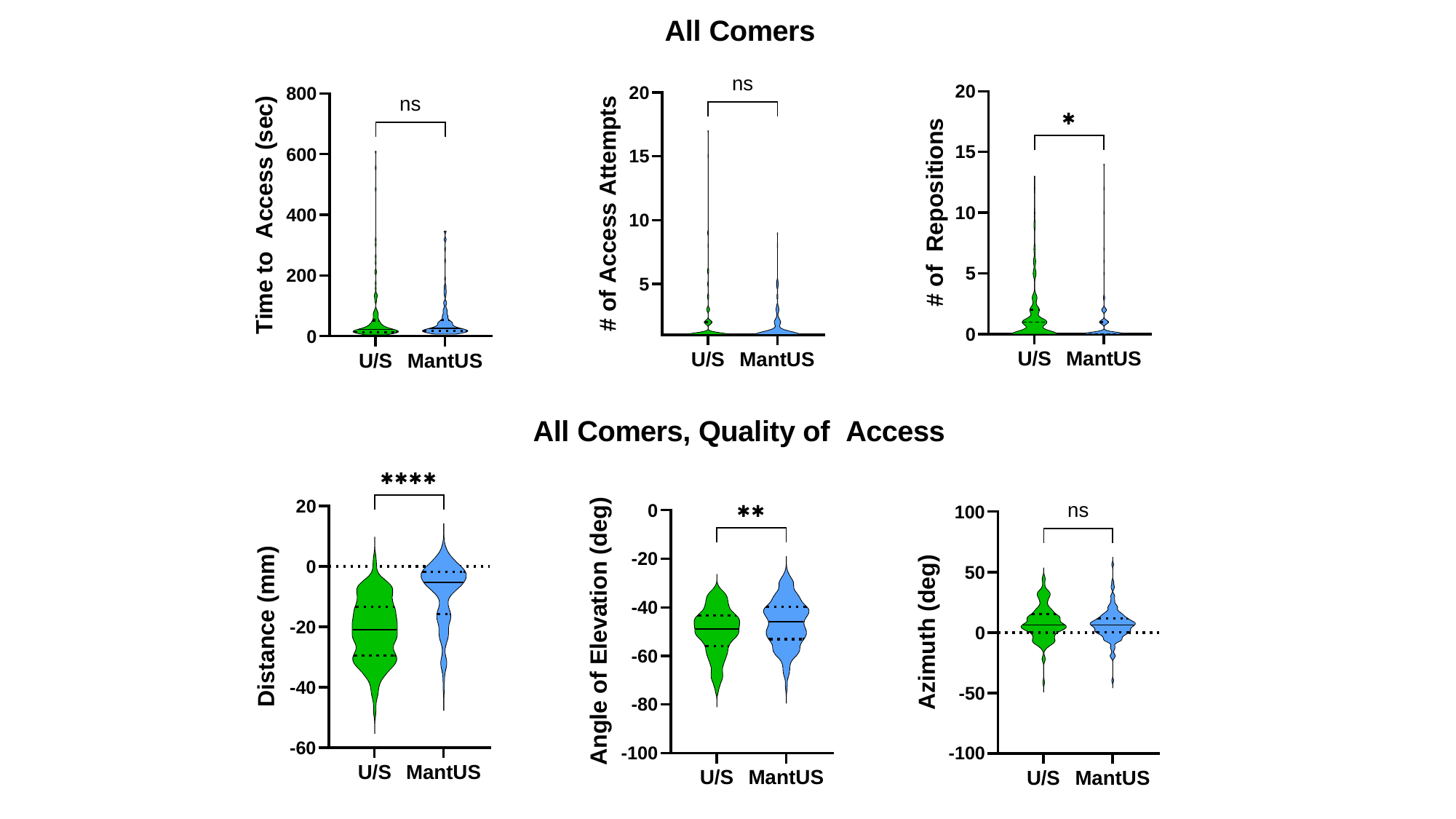

## Slide 5
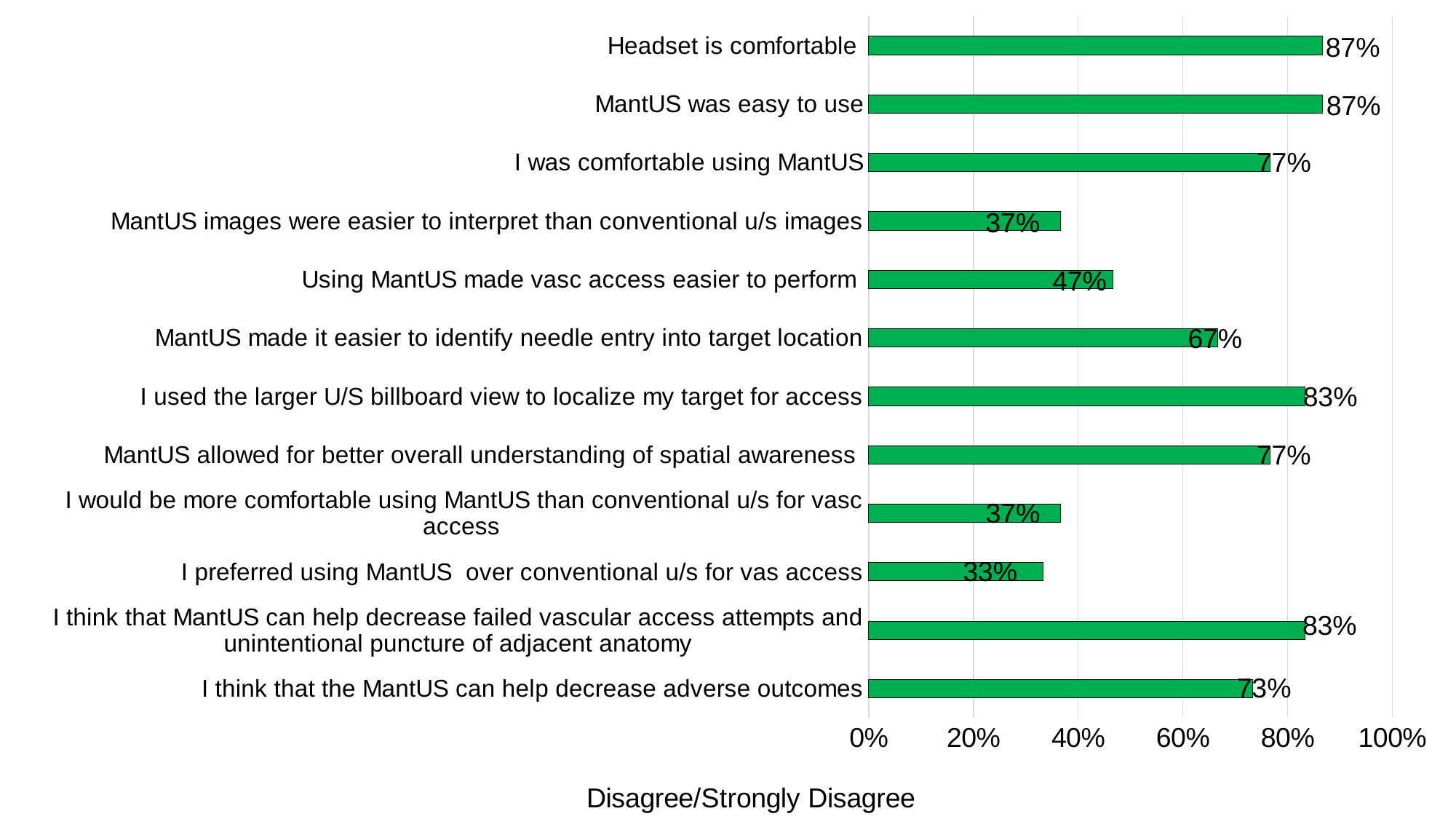

### Chart
| Category | Agree/Strongly Agree | Neutral | Disagree/Strongly Disagree |
|---|---|---|---|
| I think that the MantUS can help decrease adverse outcomes | 22.0 | 7.0 | 1.0 |
| I think that MantUS can help decrease failed vascular access attempts and unintentional puncture of adjacent anatomy | 25.0 | 4.0 | 1.0 |
| I preferred using MantUS over conventional u/s for vas access | 10.0 | 12.0 | 8.0 |
| I would be more comfortable using MantUS than conventional u/s for vasc access | 11.0 | 10.0 | 9.0 |
| MantUS allowed for better overall understanding of spatial awareness | 23.0 | 5.0 | 2.0 |
| I used the larger U/S billboard view to localize my target for access | 25.0 | 3.0 | 2.0 |
| MantUS made it easier to identify needle entry into target location | 20.0 | 8.0 | 2.0 |
| Using MantUS made vasc access easier to perform | 14.0 | 12.0 | 4.0 |
| MantUS images were easier to interpret than conventional u/s images | 11.0 | 11.0 | 8.0 |
| I was comfortable using MantUS | 23.0 | 4.0 | 3.0 |
| MantUS was easy to use | 26.0 | 3.0 | 1.0 |
| Headset is comfortable | 26.0 | 3.0 | 1.0 |87%
87%
77%
37%
47%
67%
83%
77%
37%
33%
83%
73%

## Slide 6
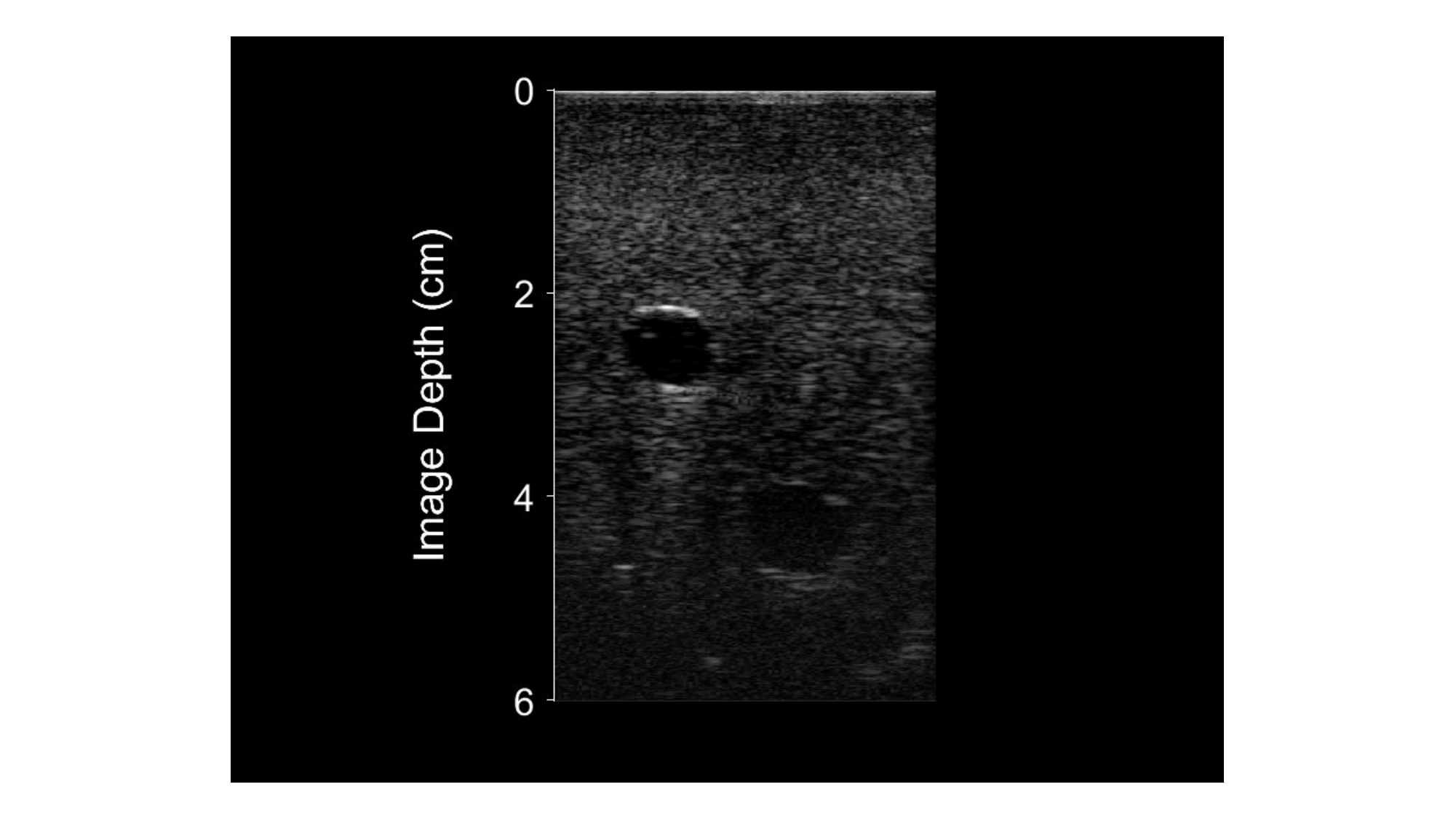

## Slide 7
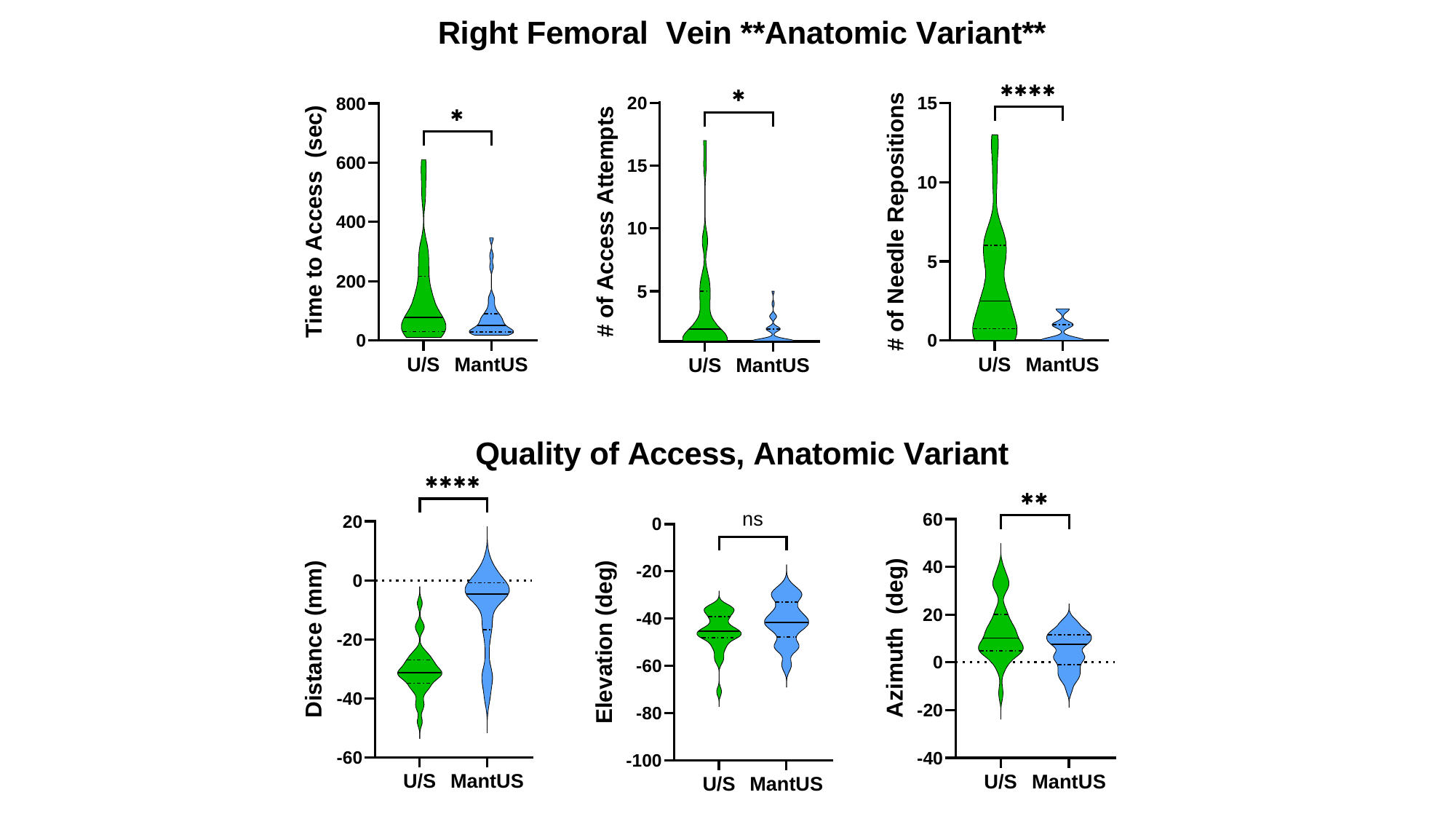

## Slide 8
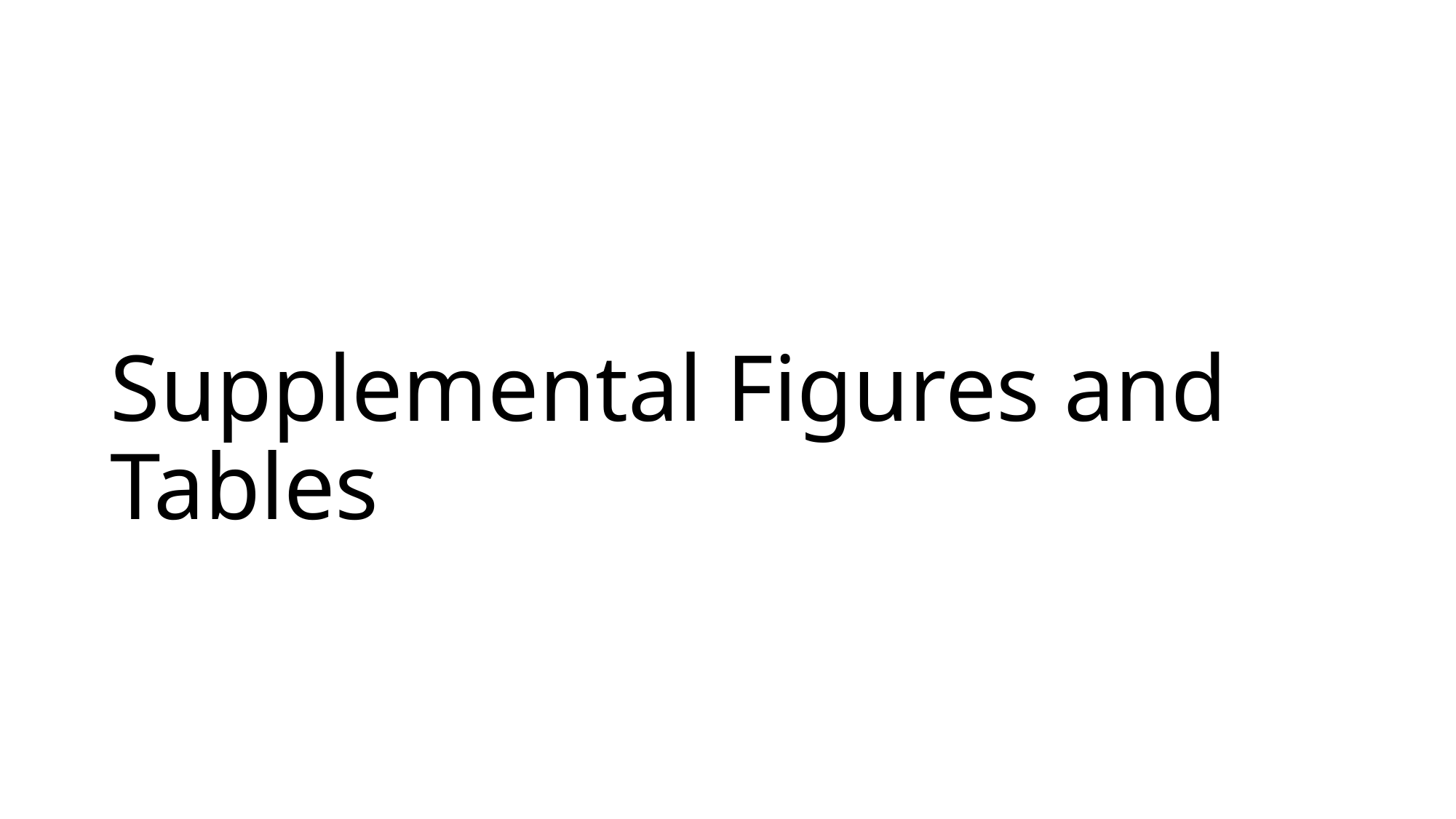

# Supplemental Figures and Tables

## Slide 9
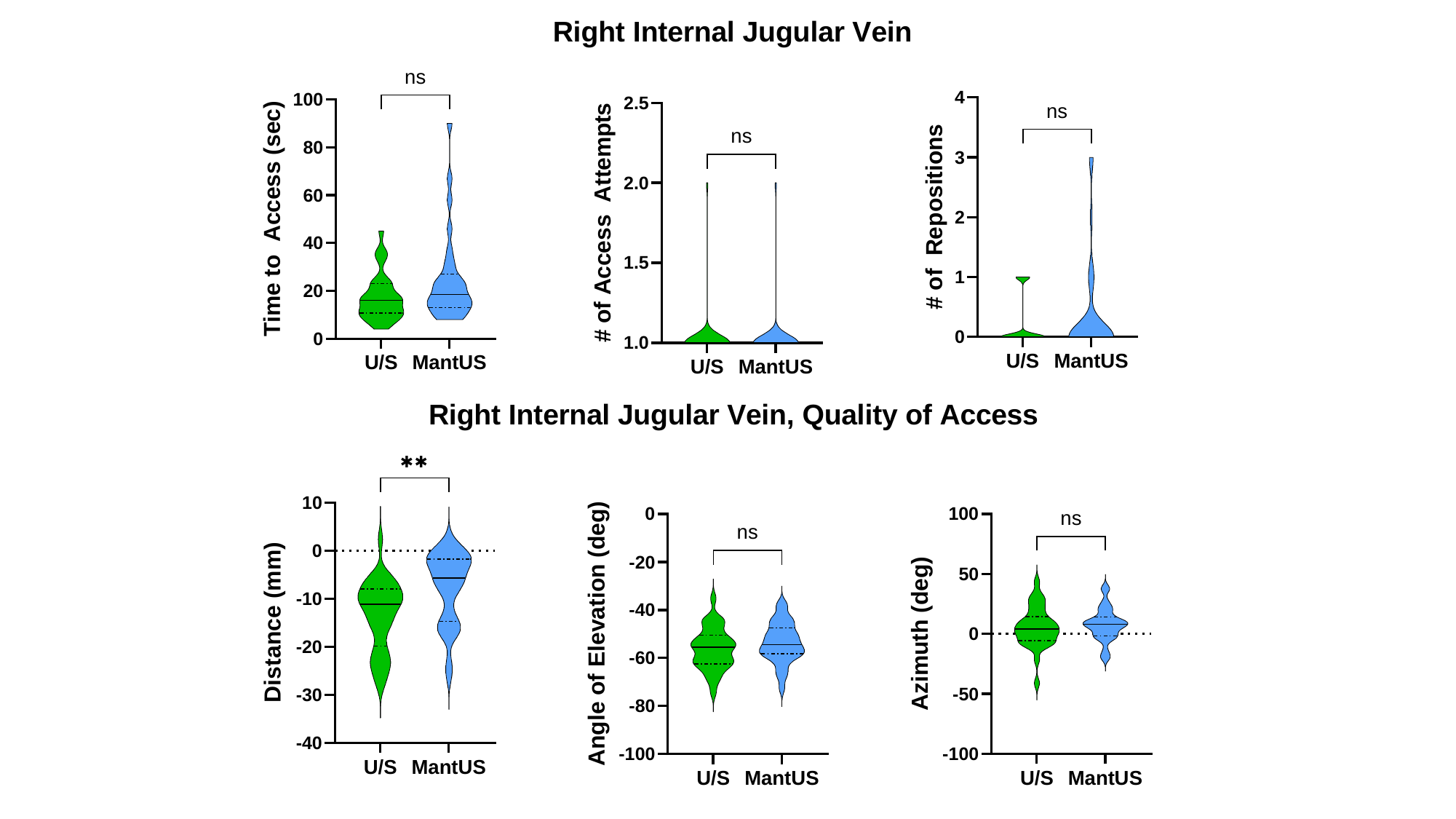

## Slide 10
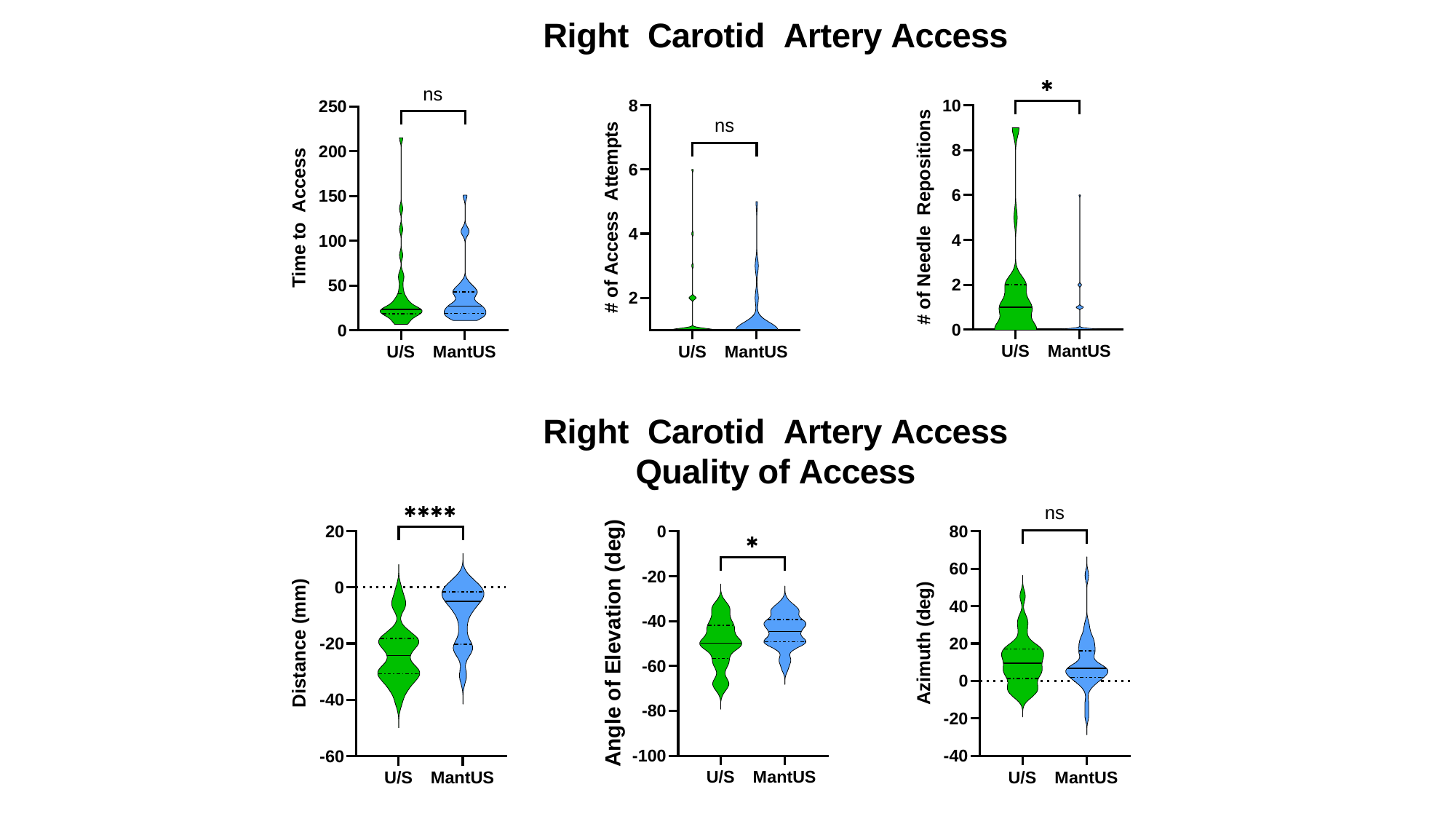

## Slide 11
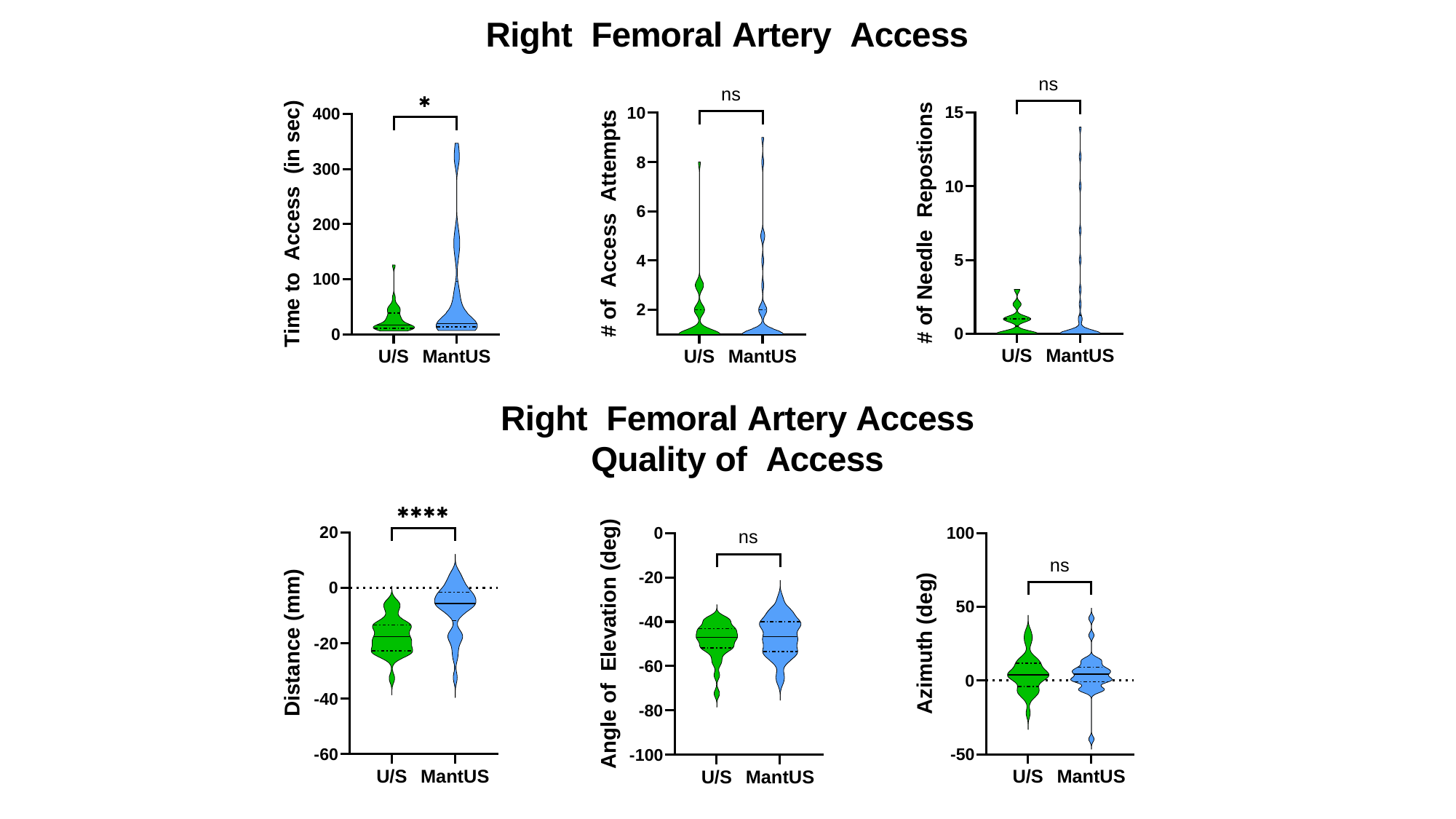

## Slide 12
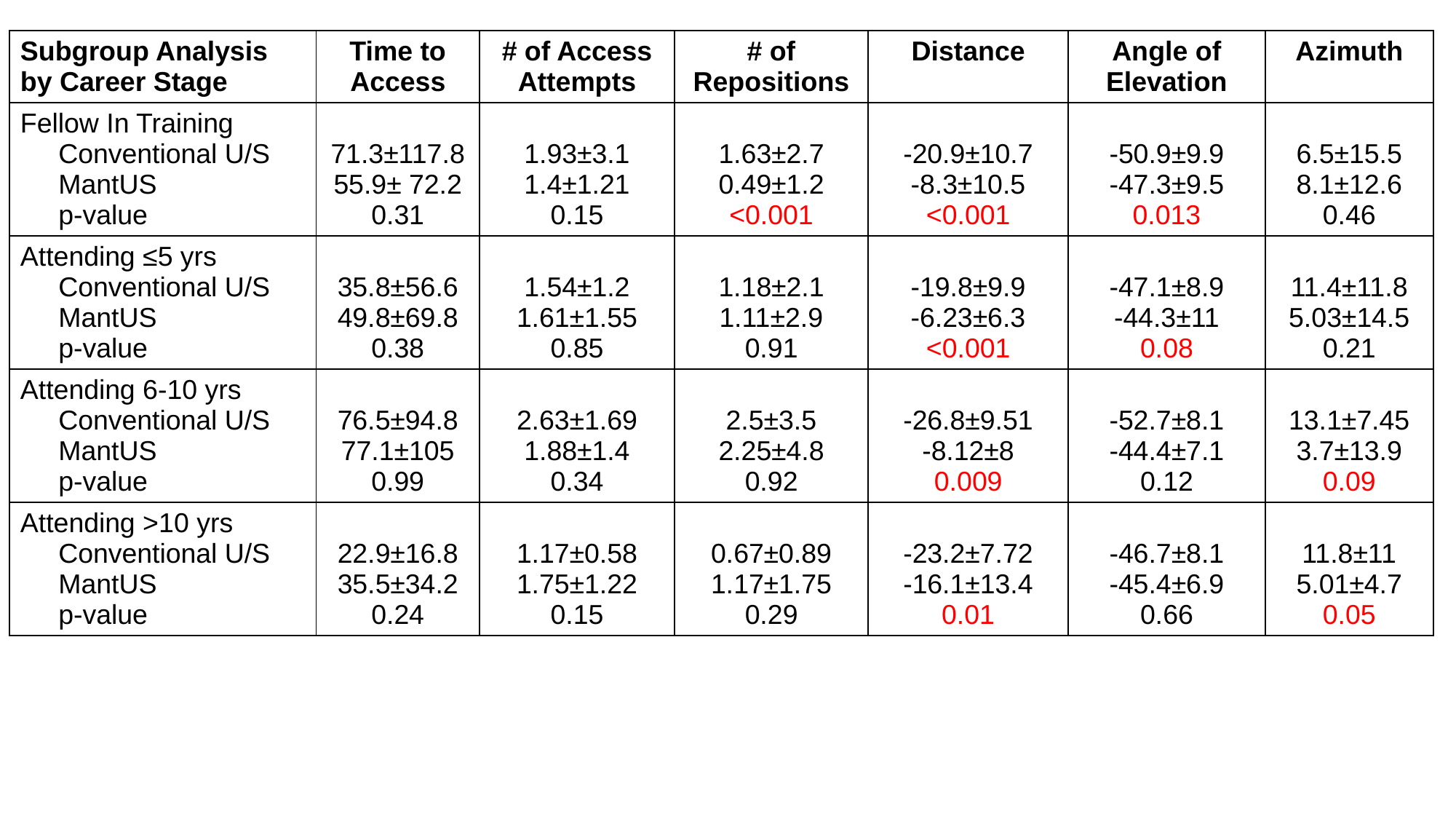

| Subgroup Analysis by Career Stage | Time to Access | # of Access Attempts | # of Repositions | Distance | Angle of Elevation | Azimuth |
| --- | --- | --- | --- | --- | --- | --- |
| Fellow In Training Conventional U/S MantUS p-value | 71.3±117.8 55.9± 72.2 0.31 | 1.93±3.1 1.4±1.21 0.15 | 1.63±2.7 0.49±1.2 <0.001 | -20.9±10.7 -8.3±10.5 <0.001 | -50.9±9.9 -47.3±9.5 0.013 | 6.5±15.5 8.1±12.6 0.46 |
| Attending ≤5 yrs Conventional U/S MantUS p-value | 35.8±56.6 49.8±69.8 0.38 | 1.54±1.2 1.61±1.55 0.85 | 1.18±2.1 1.11±2.9 0.91 | -19.8±9.9 -6.23±6.3 <0.001 | -47.1±8.9 -44.3±11 0.08 | 11.4±11.8 5.03±14.5 0.21 |
| Attending 6-10 yrs Conventional U/S MantUS p-value | 76.5±94.8 77.1±105 0.99 | 2.63±1.69 1.88±1.4 0.34 | 2.5±3.5 2.25±4.8 0.92 | -26.8±9.51 -8.12±8 0.009 | -52.7±8.1 -44.4±7.1 0.12 | 13.1±7.45 3.7±13.9 0.09 |
| Attending >10 yrs Conventional U/S MantUS p-value | 22.9±16.8 35.5±34.2 0.24 | 1.17±0.58 1.75±1.22 0.15 | 0.67±0.89 1.17±1.75 0.29 | -23.2±7.72 -16.1±13.4 0.01 | -46.7±8.1 -45.4±6.9 0.66 | 11.8±11 5.01±4.7 0.05 |

## Slide 13
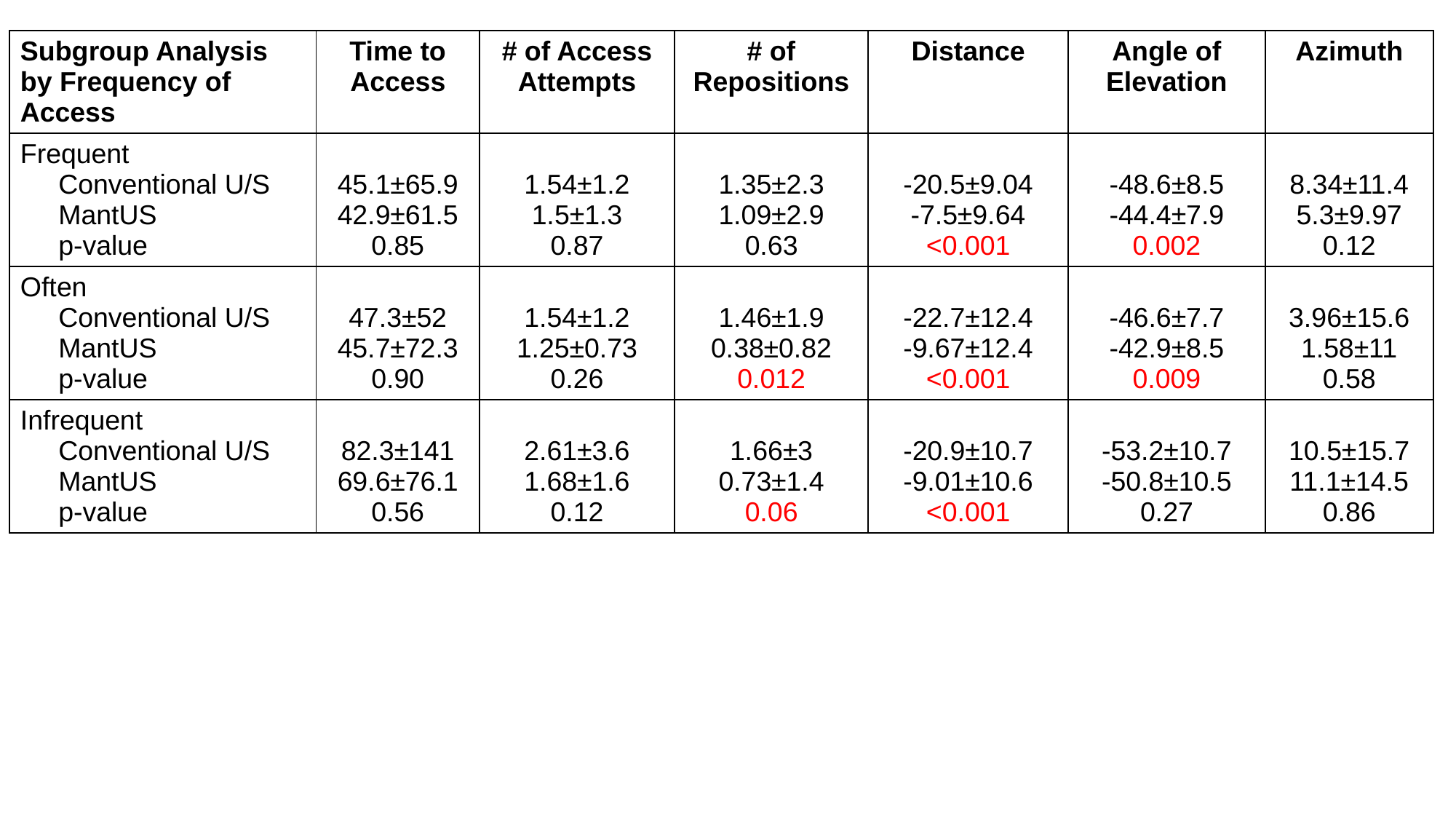

| Subgroup Analysis by Frequency of Access | Time to Access | # of Access Attempts | # of Repositions | Distance | Angle of Elevation | Azimuth |
| --- | --- | --- | --- | --- | --- | --- |
| Frequent Conventional U/S MantUS p-value | 45.1±65.9 42.9±61.5 0.85 | 1.54±1.2 1.5±1.3 0.87 | 1.35±2.3 1.09±2.9 0.63 | -20.5±9.04 -7.5±9.64 <0.001 | -48.6±8.5 -44.4±7.9 0.002 | 8.34±11.4 5.3±9.97 0.12 |
| Often Conventional U/S MantUS p-value | 47.3±52 45.7±72.3 0.90 | 1.54±1.2 1.25±0.73 0.26 | 1.46±1.9 0.38±0.82 0.012 | -22.7±12.4 -9.67±12.4 <0.001 | -46.6±7.7 -42.9±8.5 0.009 | 3.96±15.6 1.58±11 0.58 |
| Infrequent Conventional U/S MantUS p-value | 82.3±141 69.6±76.1 0.56 | 2.61±3.6 1.68±1.6 0.12 | 1.66±3 0.73±1.4 0.06 | -20.9±10.7 -9.01±10.6 <0.001 | -53.2±10.7 -50.8±10.5 0.27 | 10.5±15.7 11.1±14.5 0.86 |

## Slide 14
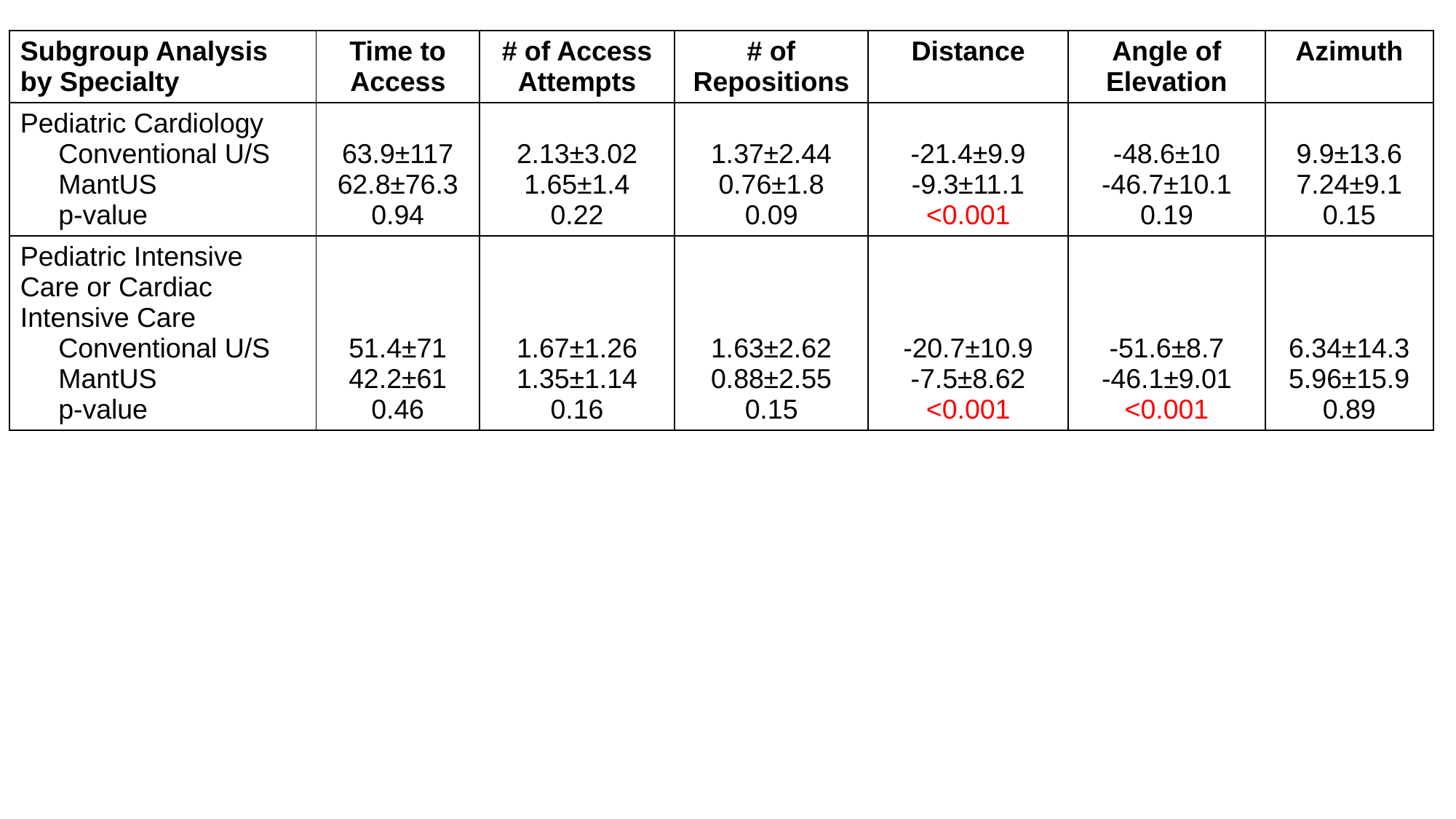

| Subgroup Analysis by Specialty | Time to Access | # of Access Attempts | # of Repositions | Distance | Angle of Elevation | Azimuth |
| --- | --- | --- | --- | --- | --- | --- |
| Pediatric Cardiology Conventional U/S MantUS p-value | 63.9±117 62.8±76.3 0.94 | 2.13±3.02 1.65±1.4 0.22 | 1.37±2.44 0.76±1.8 0.09 | -21.4±9.9 -9.3±11.1 <0.001 | -48.6±10 -46.7±10.1 0.19 | 9.9±13.6 7.24±9.1 0.15 |
| Pediatric Intensive Care or Cardiac Intensive Care Conventional U/S MantUS p-value | 51.4±71 42.2±61 0.46 | 1.67±1.26 1.35±1.14 0.16 | 1.63±2.62 0.88±2.55 0.15 | -20.7±10.9 -7.5±8.62 <0.001 | -51.6±8.7 -46.1±9.01 <0.001 | 6.34±14.3 5.96±15.9 0.89 |
