# Supplementary material for: Early preclinical experience of a mixed reality ultrasound system with active GUIDance for NEedle-based interventions: The GUIDE study
Source: Cardiovasc Digit Health J. 2022 Aug 4;3(5):232–40. doi: 10.1016/j.cvdhj.2022.07.072 (PMC9596321; doi:10.1016/j.cvdhj.2022.07.072)
Supplement: Supplemental Tables and Figures [file mmc2.docx]

**SUPPLEMENTAL TABLES AND FIGURES**

Supplemental Tables.

Supplemental Table 1. Subgroup Analysis by Career Stage. There were fewer number of needle repositions in the fellow in training group when using MantUS™ (conventional U/S 1.6±3, 0.5±1, p<0.001). Otherwise, there was no significant difference in time to access, number of access attempts or number of repositions in the other career stage groups. Using MantUS™, there was improvement in distance across all user groups by career stage (fellows in training p<0.001; attending ≤5 years p<0.001; attending 6-10 years p=0.009; attending >10 years p=0.01).

| **Subgroup Analysis by Career Stage** | **Time to Access** | **# of Access Attempts** | **# of Repositions** | **Distance** | **Angle of Elevation** | **Azimuth** |
| --- | --- | --- | --- | --- | --- | --- |
| Fellow In Training  Conventional U/S  MantUS™  p-value | 71.3±117.8  55.9± 72.2  0.31 | 1.93±3.1  1.4±1.21  0.15 | 1.63±2.7  0.49±1.2  <0.001 | -20.9±10.7  -8.3±10.5  <0.001 | -50.9±9.9  -47.3±9.5  0.013 | 6.5±15.5  8.1±12.6  0.46 |
| Attending ≤5 yrs  Conventional U/S  MantUS™  p-value | 35.8±56.6  49.8±69.8  0.38 | 1.54±1.2  1.61±1.55  0.85 | 1.18±2.1  1.11±2.9  0.91 | -19.8±9.9  -6.23±6.3  <0.001 | -47.1±8.9  -44.3±11  0.08 | 11.4±11.8  5.03±14.5  0.21 |
| Attending 6-10 yrs  Conventional U/S  MantUS™  p-value | 76.5±94.8  77.1±105  0.99 | 2.63±1.69  1.88±1.4  0.34 | 2.5±3.5  2.25±4.8  0.92 | -26.8±9.51  -8.12±8  0.009 | -52.7±8.1  -44.4±7.1  0.12 | 13.1±7.45  3.7±13.9  0.09 |
| Attending >10 yrs  Conventional U/S  MantUS™  p-value | 22.9±16.8  35.5±34.2  0.24 | 1.17±0.58  1.75±1.22  0.15 | 0.67±0.89  1.17±1.75  0.29 | -23.2±7.72  -16.1±13.4  0.01 | -46.7±8.1  -45.4±6.9  0.66 | 11.8±11  5.01±4.7  0.05 |

Supplemental Table 2. Subgroup Analysis by Frequency of Access. Users who perform vascular access often or infrequently performed better using MantUS™ as measured by number of needle repositions. Additionally, quality of access was improved across all user groups (frequent, often and infrequent) as assessed by distance. The angle of elevation was improved using MantUS™ for frequent and often access user groups.

| **Subgroup Analysis by Frequency of Access** | **Time to Access** | **# of Access Attempts** | **# of Repositions** | **Distance** | **Angle of Elevation** | **Azimuth** |
| --- | --- | --- | --- | --- | --- | --- |
| Frequent  Conventional U/S  MantUS™  p-value | 45.1±65.9  42.9±61.5  0.85 | 1.54±1.2  1.5±1.3  0.87 | 1.35±2.3  1.09±2.9  0.63 | -20.5±9.04-7.5±9.64  <0.001 | -48.6±8.5  -44.4±7.9  0.002 | 8.34±11.4  5.3±9.97  0.12 |
| Often  Conventional U/S  MantUS™  p-value | 47.3±52  45.7±72.3  0.90 | 1.54±1.2  1.25±0.73  0.26 | 1.46±1.9  0.38±0.82  0.012 | -22.7±12.4  -9.67±12.4  <0.001 | -46.6±7.7  -42.9±8.5  0.009 | 3.96±15.6  1.58±11  0.58 |
| Infrequent  Conventional U/S  MantUS™  p-value | 82.3±141  69.6±76.1  0.56 | 2.61±3.6  1.68±1.6  0.12 | 1.66±3  0.73±1.4  0.06 | -20.9±10.7  -9.01±10.6  <0.001 | -53.2±10.7  -50.8±10.5  0.27 | 10.5±15.7  11.1±14.5  0.86 |

Supplemental Table 3. Subgroup Analysis by Specialty. Study subjects from both specialties had better quality of access as measured by distance when using MantUS™ (Pediatric Cardiology p<0.001; Pediatric Intensive Care and Cardiac Intensive Care p<0.001).

| **Subgroup Analysis by Specialty** | **Time to Access** | **# of Access Attempts** | **# of Repositions** | **Distance** | **Angle of Elevation** | **Azimuth** |
| --- | --- | --- | --- | --- | --- | --- |
| Pediatric Cardiology  Conventional U/S  MantUS™  p-value | 63.9±117  62.8±76.3  0.94 | 2.13±3.02  1.65±1.4  0.22 | 1.37±2.44  0.76±1.8  0.09 | -21.4±9.9  -9.3±11.1  <0.001 | -48.6±10  -46.7±10.1  0.19 | 9.9±13.6  7.24±9.1  0.15 |
| Pediatric Intensive Care or Cardiac Intensive Care  Conventional U/S  MantUS™  p-value | 51.4±71  42.2±61  0.46 | 1.67±1.26  1.35±1.14  0.16 | 1.63±2.62  0.88±2.55  0.15 | -20.7±10.9  -7.5±8.62  <0.001 | -51.6±8.7  -46.1±9.01  <0.001 | 6.34±14.3  5.96±15.9  0.89 |

Supplemental Figures.

Supplemental Figure 1. Violin plot for right internal jugular (RIJ) venous access. When accessing the RIJ vein, there was no significant difference in time to access (conventional U/S 17±10 seconds versus MantUS™ 24±19 seconds, p=0.06), number of access attempts (conventional U/S 1±0.2 versus MantUS™ 1±0.2, p=1), or number of needle repositions (conventional U/S 0.2±0.4 versus MantUS™ 0.4±0.9 seconds, p=0.4). When assessing quality of access, there was improved distance using MantUS™ (conventional U/S -13±7 mm versus MantUS™ -8±8 mm, p=0.003) with no significant difference in angle of elevation (conventional U/S -56±9 degrees versus MantUS™ -54±8 degrees, p=0.2) or azimuth (conventional U/S 5±17 degrees versus MantUS™ 8±14 degrees, p=0.5).

Supplemental Figure 2. Violin plot for right carotid artery (RCA) access. When accessing the RCA, there was no significant difference in time to access (conventional U/S 40±45 seconds versus MantUS™ 38±32 seconds, p=0.8) or number of access attempts (conventional U/S 1.5±1 versus MantUS™ 1.3±0.9, p=0.5). There were fewer number of needle repositions noted using MantUS™ (conventional U/S 1.5±2 versus MantUS™ 0.5±1 seconds, p=0.03). When assessing quality of access, there was improvement using MantUS™ when assessing distance (conventional U/S -24±10 mm versus MantUS™ -9±10 mm, p<0.0001) and angle of elevation (conventional U/S -49±10 degrees versus MantUS™ -44±7 degrees, p=0.02). There was no significant difference in azimuth (conventional U/S 10±13 degrees versus MantUS™ 9±14 degrees, p=0.7).

Supplemental Figure 3. Violin Plot for right femoral artery (RFA) access. When accessing the RFA, there was a faster time to access using conventional U/S (conventional U/S 27±24 seconds versus MantUS™ 73±101 seconds, p=0.01), though there was no difference in number of access attempts (conventional U/S 1.7±1 versus MantUS™ 2±2, p=0.4) or number of needle repositions (conventional U/S 0.7±0.9 versus MantUS™ 1.8±3.8, p=0.1). When assessing quality of access, there was improved distance using MantUS™ (conventional U/S -17±6 mm versus MantUS™ -7±9 mm, p<0.0001) with no significant difference in angle of elevation (conventional U/S -48±8 degrees versus MantUS™ -47±9 degrees, p=0.5) or azimuth (conventional U/S 5±12 degrees versus MantUS™ 4±13 degrees, p=0.9).
